# Chromosome Duplication in *Saccharomyces cerevisiae*

**DOI:** 10.1534/genetics.115.186452

**Published:** 2016-06-27

**Authors:** Stephen P. Bell, Karim Labib

**Affiliations:** * Howard Hughes Medical Institute, Massachusetts Institute of Technology, Cambridge, Massachusetts 02139; † Medical Research Council Protein Phosphorylation and Ubiquitylation Unit, Sir James Black Centre, School of Life Sciences, University of Dundee, DD1 5EH, United Kingdom

**Keywords:** DNA replication, cell cycle, chromatin, chromosome duplication, genome stability, YeastBook

## Abstract

The accurate and complete replication of genomic DNA is essential for all life. In eukaryotic cells, the assembly of the multi-enzyme replisomes that perform replication is divided into stages that occur at distinct phases of the cell cycle. Replicative DNA helicases are loaded around origins of DNA replication exclusively during G_1_ phase. The loaded helicases are then activated during S phase and associate with the replicative DNA polymerases and other accessory proteins. The function of the resulting replisomes is monitored by checkpoint proteins that protect arrested replisomes and inhibit new initiation when replication is inhibited. The replisome also coordinates nucleosome disassembly, assembly, and the establishment of sister chromatid cohesion. Finally, when two replisomes converge they are disassembled. Studies in *Saccharomyces cerevisiae* have led the way in our understanding of these processes. Here, we review our increasingly molecular understanding of these events and their regulation.

EUKARYOTIC DNA replication requires the cell-cycle-regulated assembly of multi-enzyme replisomes that synthesize new chromosomes. These remarkable machines coordinate the action of three DNA polymerases, an RNA polymerase, and a DNA helicase to ensure the rapid, accurate, and complete replication of the eukaryotic genome. Replisome assembly starts with helicase loading during the G_1_ phase of the cell cycle and is completed during S phase when the loaded helicases are activated and DNA polymerases and many other accessory proteins are recruited. These events are facilitated by the action of an array of assembly factors. In addition, other proteins monitor the events of DNA replication and stop the process when mistakes are made to allow for DNA repair and to prevent further damage. Importantly, replisome assembly links several other processes to DNA replication including chromatin assembly and sister chromatid cohesion. Finally, a separate set of proteins including a specialized DNA polymerase, telomerase, ensures that chromosome ends are replicated and protected from damage (see Wellinger and Zakian 2012). Together, these mechanisms ensure that chromosomes are duplicated correctly and completely, and are prepared for accurate gene expression and chromosome segregation.

Several advantages have made the investigation of DNA replication in *Saccharomyces cerevisiae* particularly productive. Foremost among these is that, unlike most eukaryotic organisms, budding yeast origins of replication are defined by specific DNA sequences (Hsiao and Carbon 1979; Stinchcomb *et al.* 1979). This property has allowed yeast researchers to identify proteins that act at origins and study their function. In addition, multiple replication proteins were identified in early genetic screens, providing important footholds for replication studies (Hartwell 1976; Maine *et al.* 1984; Hennessy *et al.* 1991). Genetic-interaction studies and genome-wide analyses of the consequences of eliminating essential proteins led to the identification of additional replication factors (Kamimura *et al.* 1998, 2001; Kanemaki *et al.* 2003; Takayama *et al.* 2003). The well-understood cell cycle of *S. cerevisiae* facilitated important insights into the regulation of DNA replication initiation (Diffley 1996). Genomic approaches have also revealed the distribution of origins across the genome and their relative time of initiation in S phase (Raghuraman *et al.* 2001; Wyrick *et al.* 2001). Most recently, biochemical approaches have come to the fore. The *in vitro* reconstitution of helicase loading, helicase activation, and replication fork elongation have provided powerful insights into the major events of replication (Seki and Diffley 2000; Remus *et al.* 2009; Heller *et al.* 2011; Yeeles *et al.* 2015). Similarly, the application of structural and single-molecule studies have started to provide new levels of resolution and understanding (Sun *et al.* 2013, 2015; Ticau *et al.* 2015). Importantly, although best understood in yeast, the proteins and mechanisms of replication initiation and elongation are conserved throughout eukaryotic cells. Indeed, although this review focuses on studies of DNA replication in *S. cerevisiae*, many important contributions to our understanding of eukaryotic DNA replication emerged from studies of eukaryotic viruses (*e.g.*, SV40), other yeast (*e.g.*, *S. pombe*), and metazoan cells (particularly, the study of replication in *Xenopus* egg extracts). We refer the reader to the following collection of reviews for more information about these important studies (Bell *et al.* 2013).

In this review, we first focus on the characteristics and regulation of origins of replication. We then turn to the molecular events of replication and how these processes are coordinated with the cell cycle, monitored by checkpoint proteins, and coupled to chromatin disassembly/assembly and sister chromatid cohesion. Throughout, we emphasize the mechanistic understanding of these events in budding yeast, which has grown dramatically over the past 25 years.

## Where to Begin?

The origins of replication of *S. cerevisae* and its near relatives are defined by short 100 to 150-bp replicators (the *cis*-acting DNA sequences that direct origin function; Jacob *et al.* 1963). Knowledge of replicator location was critical to identify many replication initiation proteins, to explore replication-factor dynamics during the cell cycle, and to reveal the temporal regulation of origin usage during S phase. The defined sites of initiation also revealed the location and direction of replication forks, facilitating studies of their composition and function.

### Identification and characterization of replication origins

**Figure 1:**
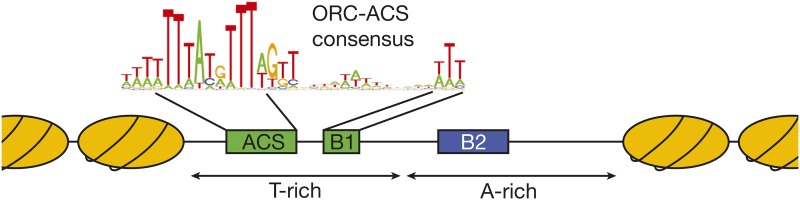
Structure of *S. cerevisiae* replicator. The general structure of budding yeast replicators and the surrounding nucleosomes is illustrated. Although the precise nucleosome positions vary, the key elements of the replicator are located within a nucleosome-free region with the ORC binding site located asymmetrically within this region. The ORC-ACS consensus sequence shown is derived from Eaton *et al.* 2010.

Replicators were originally identified by their ability to confer stable replication to episomes, and therefore called autonomously replicating sequences (ARS elements) (Stinchcomb *et al.* 1979). A subset of ARS elements was subsequently shown to act as replicators in their chromosomal locations (Brewer and Fangman 1987; Huberman *et al.* 1988). All *S. cerevisiae* replicators include an 11-bp, AT-rich, conserved sequence called the ARS consensus sequence (ACS) (Figure 1) (Broach *et al.* 1983). Further comparison of ARS elements identified an extended ACS (eACS) spanning 17 bp (Theis and Newlon 1997). The origin recognition complex (ORC; see Table 1 for a comprehensive list of proteins and complexes referred to in this review) was identified as a factor that binds *in vitro* to origin DNA in the presence of ATP, dependent upon the integrity of the ACS (Bell and Stillman 1992), and *in vivo* genomic footprinting experiments identified a very similar footprint that was regulated during the cell cycle (Diffley and Cocker 1992; Diffley *et al.* 1994). ORC is a six-protein complex, with five of the six subunits (Orc1-Orc5) being related to AAA+ ATPases (Li and Stillman 2012). Despite this similarity, only Orc1 retains ATPase activity and this subunit mediates the ATP-dependence of ORC DNA binding (Klemm *et al.* 1997). Genome-wide analysis of ORC DNA binding at high resolution identified a consensus binding site that includes the eACS but spans >30 bp, called the ORC-ACS (Xu *et al.* 2006; Eaton *et al.* 2010). Importantly, mutation of the ACS showed that this sequence is essential for replicator function in plasmids and chromosomes (reviewed in Bell 1995).

**Table 1:** Proteins and complexes referred to in this review

Protein or complex	Derivation of name	Role	Human ortholog?
Abf1	**A**RS-**b**inding **f**actor 1	Initiation: binds to the B3 element of the origin *ARS1*	?
Asf1	**A**nti-**s**ilencing **f**unction	Elongation: histone chaperone that passes newly-synthesized H3-H4 to CAF1	ASF1a/ASF1b
Cac1/Rlf2	**C**hromatin **a**ssembly **c**omplex/**R**ap1 protein **l**ocalization **f**actor	CAF1 complex; elongation: histone chaperone that deposits newly-synthesized H3-H4 onto nascent DNA	p150
Cac2	**C**hromatin **a**ssembly **c**omplex	CAF1 complex; elongation: histone chaperone that deposits newly-synthesized H3-H4 onto nascent DNA	p60
Cac3/MsiI	**C**hromatin **a**ssembly **c**omplex/**M**ulticopy **s**uppressor of **I**RA1	CAF1 complex; elongation: histone chaperone that deposits newly-synthesized H3-H4 onto nascent DNA	p48
CAF1 complex	**C**hromatin **a**ssembly **f**actor	Histone chaperone that deposits newly-synthesized H3-H4 onto nascent DNA	CAF1
Chk1	**Ch**eckpoint **k**inase	Elongation: effector protein kinase of the DNA damage checkpoint response	Functionally equivalent to CHK2, though orthologous to CHK1
Cdc6	**C**ell **d**ivision **c**ycle	Initiation: acts with ORC and Cdt1 to load Mcm2-7 helicase core	CDC6
Cdc7	**C**ell **d**ivision **c**ycle	Initiation: DDK phosphorylates Mcm2-7 to drive CMG helicase assembly	CDC7
Cdc28	**C**ell **d**ivision **c**ycle	Initiation: CDK phosphorylates Sld2 and Sld3 to drive CMG helicase assembly. Other targets too	CDK1 and CDK2
Cdc34	**C**ell **d**ivision **c**ycle	Termination: E2 ubiquitin-conjugating enzyme for SCF^Dia2^ ubiquitin ligase, required for ubiquitylation of CMG helicase	CDC34
Cdc45	**C**ell **d**ivision **c**ycle	Initiation/Elongation: subunit of CMG helicase	CDC45
Cdc48	**C**ell **d**ivision **c**ycle	Termination: AAA+ ATPase (segregase) that is required for disassembly of CMG helicase	p97
Cdc53	**C**ell **d**ivision **c**ycle	Termination: cullin subunit of SCF^Dia2^ ubiquitin ligase, required for ubiquitylation of CMG helicase	CUL1
Cdt1/TAH11/SID2	**C**dc10 **d**ependent **t**ranscription (name derived from fission yeast ortholog)	Initiation: acts with ORC and Cdc6 to load Mcm2-7 helicase core	CDT1
Chl1	**Ch**romosome **l**oss	Elongation: DNA helicase that is important for the establishment of sister chromatid cohesion	DDX11/ChLR1
Clb5 and Clb6	**C**yc**l**in **B**	Initiation: partners of Cdc28; CDK phosphorylates Sld2 and Sld3 to drive CMG helicase assembly. Other targets too	CcnB1, B2, B3 CcnA1, A2 CcnE1, and E2
CMG helicase	**C**dc45-**M**CM-**G**INS	The replicative DNA helicase, responsible for progression of replication forks	CMG
Csm3	**C**hromosome **s**egregation in **m**eiosis	RPC; elongation: Tof1-Csm3 complex binds CMG helicase and regulates aspects of fork progression	TIPIN
Ctf18/Chl12	**C**hromosome **t**ransmission **f**requency	Ctf18-RFC complex; elongation: Ctf18-RFC is important for *in vivo* level of PCNA on chromatin, binds Pol ε	CHTF18
Ctf18-RFC complex	**R**eplication **f**actor **C** (comprising Ctf18-Ctf8-Dcc1 and Rfc2-5)	Ctf18-RFC is important for *in vivo* level of PCNA on chromatin, binds Pol ε	Ctf18-RFC
Ctf19	**C**hromosome **t**ransmission **f**requency	Outer kinetochore; initiation: recruits DDK to kinetochores to mediate early firing of centromeres	CENP-P
Ctf4	**C**hromosome **t**ransmission **f**requency	RPC; elongation: adaptor that links CMG helicase to other factors at forks	AND-1/CTF4
Dbf4	**D**um**b**ell **f**ormer	Initiation: DDK, with Cdc7, phosphorylates Mcm2-7 to drive CMG helicase assembly	DBF4/ASK, DRF1
Ddc2/Lcd1	**D**NA **d**amage **c**heckpoint/**L**ethal, **c**heckpoint defective, **D**NA damage sensitive	Mec1-Ddc2 complex; elongation: protein kinase that initiates the S-phase checkpoint response	ATRIP
Dia2	**D**igs **i**nto **a**gar	Termination: F-box protein, subunit of SCF^Dia2^ ubiquitin ligase, required for ubiquitylation and disassembly of CMG helicase	Orthologs only identified in yeasts, so another E3 ubiquitin ligase might play a similar role in higher eukaryotes.
Dls1	**D**pb3-Like Subunit of ISW2/yCHRAC complex	Chromatin remodeling; component of yCHRAC complex	CHRAC1
Dna2	**DNA** synthesis defective	Elongation: nuclease/helicase that cuts long flaps, generated when Pol δ displaces 5′ end of preceding Okazaki fragment	DNA2
Dpb2	**D**NA **p**olymerase **B** subunit 2	Pol ε complex, B subunit; initiation/elongation: Dpb2 is required for GINS recruitment to origins, and is also needed to tether Pol ε to the CMG helicase at forks	Pole2/p59
Dpb3	**D**NA **p**olymerase **B** subunit 3	Pol ε complex, B subunit; initiation/elongation: Dpb3-Dpb4 bind dsDNA and have a histone fold	Pole3/p17
Dpb4	**D**NA **p**olymerase **B** subunit 4	Pol ε complex, B subunit; initiation/elongation: Dpb3-Dpb4 bind dsDNA and have a histone fold	Pole4/p12
Eco1/Ctf7	**E**stablishment of **co**hesion	Elongation: acetyltransferase that modifies cohesin and is importance for establishment of sister chromatid cohesion	ESCO2
Elg1	**E**nhanced **l**evel of **g**enomic instability	Elg1-RFC complex; elongation: Elg1-RFC unloads PCNA from replication forks	Elg1
Elg1-RFC complex	**R**eplication **f**actor **C** (comprising Elg1 and Rfc2-5)	Elg1-RFC unloads PCNA from replication forks	Elg1-RFC
FACT complex	**Fa**cilitates **c**hromatin **t**ransactions	Histone chaperone comprising Spt16 and Pob3; forms part of RPC around the CMG helicase	FACT
Fen1/Rad27/Erc11	**F**lap structure-specific **en**donuclease/**rad**iation sensitive	Elongation: nuclease that cuts short flaps during processing of Okazaki fragments	FEN1
Fkh1	**F**or**kh**ead homolog	Initiation: transcription factor that promotes early firing of some origins of replication	Forkhead family of transcription factors
Fkh2	**F**or**kh**ead homolog	Initiation: transcription factor that promotes early firing of some origins of replication	Forkhead family of transcription factors
GINS complex	**G**o-**I**chi-**N**ii-**S**an (Japanese for 5-1-2-3, corresponding to numbers at end of names of Sld5/Cdc105-Psf1/Cdc101-Psf2/Cdc102-Psf3)	Essential component of the CMG helicase at replication forks	GINS
Glc7/CID1/DIS2/PP1/DIS2S1	**Gl**y**c**ogen	Initiation: type 1 protein phosphatase that counteracts DDK activity at origins	PP1
Hrt1	**H**igh level expression **r**educes **T**y3 transposition	Termination: RING subunit of SCF^Dia2^ ubiquitin ligase	RBX1
Htz1	**H**istone Two A Z1	Histone variant H2AZ; role in transcriptional regulation, preventing spread of heterochromatin	H2A.Z
Mcm2-7 complex	**M**ini**c**hromosome **m**aintenance	Catalytic core of the CMG helicase	Mcm2-7 complex
Mcm2	**M**ini**c**hromosome **m**aintenance	Mcm2-7 complex; initiation/elongation: catalytic core of CMG helicase	MCM2
Mcm3	**M**ini**c**hromosome **m**aintenance	Mcm2-7 complex; initiation/elongation: catalytic core of CMG helicase	MCM3
Mcm4/Cdc54	**M**ini**c**hromosome **m**aintenance	Mcm2-7 complex; initiation/elongation: catalytic core of CMG helicase	MCM4
Mcm5/Cdc46/Bob1	**M**ini**c**hromosome **m**aintenance	Mcm2-7 complex; initiation/elongation: catalytic core of CMG helicase	MCM5
Mcm6	**M**ini**c**hromosome **m**aintenance	Mcm2-7 complex; initiation/elongation: catalytic core of CMG helicase	MCM6
Mcm7/Cdc47	**M**ini**c**hromosome **m**aintenance	Mcm2-7 complex; initiation/elongation: catalytic core of CMG helicase	MCM7
Mcm10/Dna43	**M**ini**c**hromosome **m**aintenance	Initiation (Elongation?): activation of CMG helicase	MCM10
Mec1/Esr1/Sad3	**M**itosis **e**ntry **c**heckpoint	Mec1-Ddc2 complex; elongation: protein kinase that initiates the S-phase checkpoint response	ATR
Mlh1/Pms2	**M**ut**L** **h**omolog	Forms complex with Pms1 and Msh2-Msh3; elongation: is important for mismatch repair	MLH1
Mlh2	**M**ut**L** **h**omolog	Forms complex with Mlh1; elongation: plays a role in mismatch repair	PMS1
Mlh3	**M**ut**L** **h**omolog	Forms complex with Mlh1; elongation: plays a role in mismatch repair	MLH3
Mms2	**M**ethyl **m**ethane**s**ulfonate sensitivity	Mms2-Ubc13 complex; elongation: E2 ubiquitin-conjugating enzymes that work with Rad5 to polyubiquitylate PCNA, after DNA damage	MMS2
Mrc1	**M**ediator of the **r**eplication **c**heckpoint	Elongation: required downstream of Mec1 to activate the Rad53 S-phase checkpoint kinase, also important for normal fork progression	CLASPIN
Msh2	**M**ut**S** **h**omolog	Msh complex; elongation: binds to DNA mismatches and is important for mismatch repair	MSH2
Msh3	**M**ut**S** **h**omolog	Msh complex; elongation: binds to Msh2 and is important for mismatch repair	MSH3
Msh6	**M**ut**S** **h**omolog	Msh complex; elongation: binds to Msh2 and is important for mismatch repair	MSH6
ORC	**O**rigin **r**ecognition **c**omplex (Orc1-6)	Binds to origin DNA and acts with Cdc6 and Cdt1 to load Mcm2-7 helicase core	ORC
Orc1	**O**rigin **r**ecognition **c**omplex	ORC complex; initiation: loads Mcm2-7 helicase core	ORC1
Orc2	**O**rigin **r**ecognition **c**omplex	ORC complex; initiation: loads Mcm2-7 helicase core	ORC2
Orc3	**O**rigin **r**ecognition **c**omplex	ORC complex; initiation: loads Mcm2-7 helicase core	ORC3
Orc4	**O**rigin **r**ecognition **c**omplex	ORC complex; initiation: loads Mcm2-7 helicase core	ORC4
Orc5	**O**rigin **r**ecognition **c**omplex	ORC complex; initiation: loads Mcm2-7 helicase core	ORC5
Orc6	**O**rigin **r**ecognition **c**omplex	ORC complex; initiation: loads Mcm2-7 helicase core	ORC6
Pds5	**P**recocious Dissociation of Sisters	Associates with cohesin complex and preserves its integrity	PDS5A, PDS5B
Pif1	**P**etite **i**ntegration **f**requency	Elongation: DNA helicase related to Rrm3, important for forks to pass through G4 quadruplex DNA and past protein–DNA barriers	PIF1
Pms1	**P**ost**m**eiotic **s**egregation	Forms heterodimer with Mlh1; elongation: binds DNA and is important for mismatch repair	PMS2
Pol1/Cdc17/Crt5/Lrs9/Hpr3	**Pol**ymerase	Pol α complex, polymerase subunit; initiation/elongation: Pol α makes RNA-DNA primers for leading-/lagging-strand synthesis	PolA/p180
Pol2/Dun2	**Pol**ymerase	Pol ε complex, polymerase subunit; initiation/elongation: Pol ε is required for GINS recruitment to origins and thus for CMG assembly, it then extends the leading strand at forks	Pole/p261
Pol3/Cdc2	**Pol**ymerase	Pol δ complex, polymerase subunit; elongation: Pol δ extends Okazaki fragments during lagging-strand synthesis	Pold1/p125
Pol12	**Pol**ymerase	Pol α complex, B subunit; initiation/elongation: Pol α makes RNA-DNA primers for leading-/lagging-strand synthesis	PolA2/p68
Pol30	**Pol**ymerase	PCNA; elongation: processivity clamp for Pol δ	PCNA
Pol31/Hys2/Hus2/Sdp5	**Pol**ymerase	Pol δ complex, B subunit; elongation: Pol δ extends Okazaki fragments during lagging-strand synthesis	Pold2/p50
Pol32	**Pol**ymerase	Pol δ complex, smallest subunit; elongation: Pol δ extends Okazaki fragments during lagging-strand synthesis	Pold3/p66
Pob3	**Po**l1 **b**inding	FACT complex; elongation: histone chaperone that forms part of the RPC at replication forks	SSRP1
Pri1	DNA **pri**mase	Pol α complex, primase subunit; initiation/elongation: Pol α makes RNA-DNA primers for leading-/lagging-strand synthesis	Prim1/p48
Pri2	DNA **pri**mase	Pol α complex, primase subunit; initiation/elongation: Pol α makes RNA-DNA primers for leading-/lagging-strand synthesis	Prim2/p58
Psf1/Cdc101	**P**artner of **S**ld **f**ive (Sld5)	Initiation/Elongation: subunit of GINS complex, and thus of CMG helicase	PSF1/GINS1
Psf2/Cdc102	**P**artner of **S**ld **f**ive (Sld5)	Initiation/Elongation: subunit of GINS complex, and thus of CMG helicase	PSF2/GINS2
Psf3	**P**artner of **S**ld **f**ive (Sld5)	Initiation/Elongation: subunit of GINS complex, and thus of CMG helicase	PSF3/GINS3
Rad5	**Rad**iation sensitive	Elongation: E3 ubiquitin ligase that works with Mms2-Ubc13 to polyubiquitylate PCNA, after DNA damage	HLTF
Rad6	**Rad**iation sensitive	Elongation: ubiquitin-conjugating enzyme that works with Rad18 to mono-ubiquitylate PCNA, after DNA damage	RAD6
Rad30	**Rad**iation sensitive	Elongation: translesion DNA polymerase (Pol η)	Pol η
Rad53/Lsd1/Mec2/Spk1	**Rad**iation sensitive	Elongation: effector protein kinase of the S-phase checkpoint response	Functionally equivalent to CHK1, though orthologous to CHK2
Rad61/Wpl1	**Rad**iation sensitive	Elongation: destablizes cohesin ring and thus antagonizes the establishment of sister chromatid cohesion	Wapl
Rev3	**Rev**ersionless	Elongation: translesion DNA polymerase (subunit of Pol ζ)	Pol ζ
Rev7	**Rev**ersionless	Elongation: translesion DNA polymerase (subunit of Pol ζ)	Pol ζ
Rfa1/Buf2/Fun3/Rpa1	**R**eplication **f**actor **A** (the name comes from studies of SV40 DNA replication)	RPA complex; initiation/elongation: RPA coats ssDNA at replication forks	RPA1/p70
Rfa2/Buf1/Rpa2	**R**eplication **f**actor **A** (the name comes from studies of SV40 DNA replication)	RPA complex; initiation/elongation: RPA coats ssDNA at replication forks	RPA2/^32^P
Rfa3	**R**eplication **f**actor **A** (the name comes from studies of SV40 DNA replication)	RPA complex; initiation/elongation: RPA coats ssDNA at replication forks	RPA3/p14
Rfc1-RFC complex	**R**eplication **f**actor **C** (comprising Rfc1-5; the name comes from studies of SV40 DNA replication)	Rfc1-RFC binds to 3′ end of primers bound to template and loads PCNA around dsDNA	RFC
Rfc1/Cdc44	**R**eplication **f**actor **C** (the name comes from studies of SV40 DNA replication)	RFC complex; elongation: Rfc1-RFC binds to 3′ end of primers bound to template and loads PCNA around dsDNA	Rfc1/p140
Rfc2	**R**eplication **f**actor **C** (the name comes from studies of SV40 DNA replication)	RFC complex; elongation	Rfc2/p40
Rfc3	**R**eplication **f**actor **C** (the name comes from studies of SV40 DNA replication)	RFC complex; elongation	Rfc3/p38
Rfc4	**R**eplication **f**actor **C** (the name comes from studies of SV40 DNA replication)	RFC complex; elongation	Rfc4/p37
Rfc5	**R**eplication **f**actor **C** (the name comes from studies of SV40 DNA replication)	RFC complex; elongation	Rfc5/p36
Rif1	**R**AP1-**i**nteracting **f**actor	Initiation: delays origin firing by recruitment of Glc7 protein phosphatase	RIF1
RPA	**R**eplication **p**rotein **A** (comprising Rfa1-Rfa3; the names come from studies of SV40 DNA replication)	The eukaryotic ssDNA binding complex at replication forks	RPA
RPC	**R**eplisome **p**rogression **c**omplex (CMG, Ctf4, Tof1-Csm3, Mrc1, FACT, and Top1)	Assembles around the CMG helicase at forks. The RPC associates with Pol ε, Pol α and SCF^Dia2^	RPC
Rpd3	**R**educed **p**otassium **d**ependency	Initiation: histone deacetylase; particularly important for regulation of origins in rDNA	RPD3
Rrm3	**r**DNA **r**ecombination **m**utation	Elongation: DNA helicase related to Pif1; important for forks to pass protein–DNA barriers	PIF1
Rtt101	**R**egulator of **T**y1 **t**ransposition	Elongation: cullin that forms an E3 ligase important for survival of DNA damage	STAG1-3
Rtt106	**R**egulator of **T**y1 **t**ransposition	Elongation: histone chaperone that deposits newly-synthesized H3-H4 onto DNA	STAG1-3
Rtt109	**R**egulator of **T**y1 **t**ransposition	Elongation: histone acetyltransferase that acetylates K56 of histone H3	STAG1-3
Scc3	**S**ister Chromatid Cohesion	Component of cohesin complex; maintains sister chromatid cohesion until mitosis	
SCF complex	**S**kp1-Cullin-F-box protein	Cullin 1 ubiquitin ligase, in which substrate binding is mediated by F-box proteins	SCF
Sgs1	**S**low Growth Suppressor (referring to suppression of the growth defect of top3Δ)	Elongation: yeast ortholog of Bloom DNA helicase, processes recombination intermediates	Bloom helicase
Sir3	**S**ilent **i**nformation **r**egulator	Sir complex; initiation: required to maintain transcriptionallysilent chromatin at telomeres	?
Sic1	**S**ubstrate/Subunit Inhibitor of Cyclin-dependent protein kinase	Cell cycle control; inhibitor of B-cyclin associated Cdc28 kinase	?
Siz1	**S**AP and m**IZ**-finger domain	Elongation: E3 SUMO ligase that works with Ubc9 to sumoylate PCNA	PIAS4
Skp1	**S**uppressor of **k**inetochore **p**rotein mutant	Termination: adaptor subunit of SCF^Dia2^ ubiquitin ligase, required for ubiquitylation and disassembly of CMG helicase	SKP1
Sld2/Drc1	**S**ynthetic **l**ethal with **d**pb11-1	Initiation: assembly of CMG helicase	RECQL4
Sld3	**S**ynthetic **l**ethal with **d**pb11-1	Initiation: assembly of CMG helicase	Treslin/TICRR
Sld5 / Cdc105	**S**ynthetic **l**ethal with **d**pb11-1	Initiation/Elongation: subunit of GINS complex, and thus of CMG helicase	SLD5/GINS4
Sld7	**S**ynthetic **l**ethal with **d**pb11-1	Partner of Sld3; initiation: assembly of CMG helicase	?
Smc5	**S**tructural **m**aintenance of **c**hromosomes	Smc5-Smc6 complex (with other factors); elongation: key role in removal of X-shaped structures that arise between sister chromatids during replication, the complex has associated SUMO ligase activity	SMC5
Smc6	**S**tructural **m**aintenance of **c**hromosomes	Smc5-Smc6 complex (with other factors); elongation: key role in removal of X-shaped structures that arise between sister chromatids during replication, the complex has associated SUMO ligase activity	SMC6
Spt16	**S**u**pp**ressor of **T**y	FACT complex; elongation: histone chaperone that forms part of the RPC at replication forks	SUPT16H
Srs2	**S**uppressor of **R**ad **s**ix	Elongation: DNA helicase that is recruited to forks by sumoylated PCNA and disassembles Rad51 filaments	RTEL1
Tof1	**To**poisomerase I interacting **f**actor	RPC; elongation: Tof1-Csm3 complex binds CMG helicase and regulates aspects of fork progression	TIMELESS
Top1	**Top**oisomerase I	Elongation: topoisomerase I	Top1/Topo I
Top2	**Top**oisomerase II	Elongation: topoisomerase II	Top2/Topo II
Ubc9	**Ub**iquitin **c**onjugating	Elongation: E2 SUMO-conjugating enzyme that works with Siz1 to sumoylate PCNA	UBC9/UBE2I
Ubc13	**Ub**iquitin **c**onjugating	Mms2-Ubc13 complex; elongation: E2 ubiquitin-conjugating enzymes that works with Rad5 to polyubiquitylate PCNA, after DNA damage	UBC13
Vps75	**R**egulator of **T**y1 **t**ransposition	Elongation: histone chaperone that deposits newly-synthesized H3-H4 onto DNA	SET?
yCHRAC	**Y**east Chromatin accessibility complex (Isw2, Itc1, Dls1, Dpb4)	Chromatin remodeling	CHRAC

For each factor, the table shows the derivation of the name, a brief summary of the factor’s role, and the human ortholog if known.

Mutagenesis of the *ARS1* replicator revealed that sequences located 3′ to the T-rich strand of the ACS are also required to direct replication initiation (Marahrens and Stillman 1992; Liachko *et al.* 2010). Mutants in any of three elements (B1, B2, and B3) reduce origin activity but when mutated simultaneously, they eliminate origin function. Along with the ACS, the B1 element is part of the ORC-ACS binding site (Rao and Stillman 1995; Rowley *et al.* 1995), although B1 may have additional functions during helicase loading (Speck and Stillman 2007). The B2 element frequently resembles an inverted ACS (Wilmes and Bell 2002; Liachko *et al.* 2010) but shorter A-rich sequences unrelated to the ACS can also function (Chang *et al.* 2011). Functional analysis shows the B2 element facilitates helicase loading after ORC DNA binding (Zou and Stillman 2000; Lipford and Bell 2001). The B3 element is a binding site for Abf1, which acts to position nucleosomes adjacent to the origin (Lipford and Bell 2001). Only the B1 element shows sequence conservation in other origins (as part of the ORC-ACS). Nevertheless, functional equivalents to the B2 element have been identified at other replicators (Rao *et al.* 1994; Theis and Newlon 1994) and binding sites for Abf1 and other nucleosome positioning proteins have been identified at a subset of origins (Buchman *et al.* 1988).

Although both the ACS and the B regions are AT-rich, they show a strong but opposite bias for T residues on one strand. Thus, the DNA strand that is T-rich within the ACS is highly A-rich in the B region (Figure 1) and this bias has been exploited to identify origins (Breier *et al.* 2004). A-rich regions are known to be strong nucleosome-excluding signals, and this bias may contribute to the nucleosome-free nature of origins (Breier *et al.* 2004; Berbenetz *et al.* 2010; Eaton *et al.* 2010).

### Genome-wide studies of DNA replication

Several approaches have been used to identify origins across the yeast genome (reviewed in MacAlpine and Bell 2005). The most direct methods (called replication-timing profiles) used synchronized cell populations to identify the relative time of replication of all segments of the genome (Raghuraman *et al.* 2001; Yabuki *et al.* 2002). Because origin DNA will, by definition, replicate before the surrounding DNA sequences, these sequences appear as local minima of replication times. Genome-wide analysis of chromatin immunoprecipitation (ChIP) of the catalytic core of the replicative helicase during the G_1_ phase of the cell cycle also reveals origin DNA sequences (Wyrick *et al.* 1999; Xu *et al.* 2006; Eaton *et al.* 2010). Because all origins must load the helicase core during G_1_, sites of helicase localization identify potential origins of replication. Strand-specific deep sequencing of Okazaki fragments maps origins by identifying the change in the strand bias of Okazaki fragments that occurs at origins of replication (McGuffee *et al.* 2013). In addition, the original plasmid-based method to identify ARS elements has been combined with deep sequencing to comprehensively identify short sequences that act as replicators on plasmids (Liachko *et al.* 2013).

Genome-wide views of DNA replication have revealed important attributes of yeast replication origins and their regulation. Replication-timing profiles revealed a temporal order of DNA replication across the genome and showed that yeast origins are consistently bidirectional (Raghuraman *et al.* 2001). Origins of similar timing cluster along the chromosomes (Yabuki *et al.* 2002); origins near centromeres are early replicating and those near telomeres are late replicating (see below). The higher resolution of ChIP studies showed that the majority of origins are located in intergenic regions (Wyrick *et al.* 1999; Eaton *et al.* 2010). Finally, sequencing of Okazaki fragments provided information that allows the separate determination of origin efficiency and replication timing (McGuffee *et al.* 2013).

The total number of origins identified by these approaches varies; however, data from many studies has been used to create a database of *S. cerevisiae* origins, called OriDB (Siow *et al.* 2012). Currently, OriDB identifies >600 “confirmed” or “likely” origins. Because repeated sequences are included only once in the database, this number of potential origins is an underestimate. Each of the ∼150 ribosomal DNA (rDNA) repeats found on chromosome XII includes an origin, although in wild-type cells only ∼25% of these initiate in any cell cycle (Pasero *et al.* 2002). Similarly, the X and Y′ telomeric repeat sequences are known to contain functional origin sequences (Chan and Tye 1983). Although these numbers represent an accounting of all potential origins, many origins initiate in <50% of cell divisions (for example, Friedman *et al.* 1997). Thus, in any given cell cycle only a subset of the >700 potential origins will initiate replication. The remaining origins are inactivated by replisomes derived from adjacent origins (Santocanale *et al.* 1999; Vujcic *et al.* 1999). The excess of origins likely act as “backup” initiation sites if replication forks from adjacent origins encounter difficulties, as has been proposed in vertebrate cells (Ge *et al.* 2007).

The sites and activity of budding yeast origins of replication are largely the same in all cell types and under different growth conditions. One exception is the small subset of origins of replication that are contained within transcribed regions of the genome. There are ∼35 origins of replication that load helicases and initiate replication specifically in mitotic or meiotic cells (Mori and Shirahige 2007; Blitzblau *et al.* 2012). The majority of these origins are located within genes that are only transcribed in mitotic or meiotic cells and are only active when the gene they are contained within is inactive.

### Local chromatin structure influences origin selection and function

In addition to the ACS, local nucleosome positioning also influences origin selection. There are many more matches to the ORC-ACS than sites of ORC binding in the yeast genome (Eaton *et al.* 2010). Mapping of nucleosome location across the yeast genome revealed that the bound ORC-ACS sites are typically within a nucleosome-free region (NFR) flanked by positioned nucleosomes on either side (Figure 1) (Berbenetz *et al.* 2010; Eaton *et al.* 2010). Thus, the presence of overlapping nucleosomes at the unbound ORC-ACS sites suggests that these nucleosomes inhibit ORC binding. Analysis of cells in which ORC DNA binding was inactivated shows that a smaller NFR is still found without an ORC, providing ORC access to the ACS. The A-rich nature of the origin sequences, which are known to be poor sites for nucleosome formation (Segal and Widom 2009), is likely responsible for the lack of origin-associated nucleosomes.

The nucleosomes that flank origins of replication are more dynamic than the average nucleosomes (Dion *et al.* 2007) and are enriched for the yeast H2A.Z variant histone known as Htz1 (Albert *et al.* 2007). The observed dynamism is not due to the events of replication initiation as it is observed in cells arrested in G_1_ (Albert *et al.* 2007). Cell-cycle studies of origin-proximal nucleosomes found that efficient origins expand the NFR at the origin during G_1_, most likely as a consequence of helicase loading (Belsky *et al.* 2015). Interestingly, mutations in the SWI/SNF nucleosome-remodeling complex cause defects in origin function, although it is unclear if these effects are direct (Flanagan and Peterson 1999).

Consistent with an important role of proximal nucleosomes, changing the position of local nucleosomes inhibits origin function. Moving the ORC-adjacent nucleosome at *ARS1* closer to the origin (into the NFR) dramatically inhibits plasmid stability (Simpson 1990), presumably by interfering with ORC DNA binding. ORC is responsible for positioning this nucleosome, and moving it away from the origin also inhibits replication initiation by reducing helicase loading (Lipford and Bell 2001).

## Many are Called: the Principles of Helicase Loading

Although initial origin recognition is mediated by ORC, loading of the replicative DNA helicase is required to mark a site as a potential origin of replication and is referred to as replication origin licensing (Blow and Laskey 1988). This event was initially characterized as a G_1_-specific change in the *in vivo* footprint at yeast origins of replication referred to as prereplicative complex formation (Diffley *et al.* 1994) and was subsequently shown to reflect helicase loading (Labib *et al.* 2001). Restricting helicase loading to G_1_ is essential to ensure that the eukaryotic genome is replicated once per cell cycle (Siddiqui *et al.* 2013).

### Mcm2-7 is loaded around origin DNA during G1-phase

The core enzyme of the eukaryotic replicative DNA helicase is the Mcm2-7 complex. The six Mcm2-7 proteins were identified in two genetic screens in yeast and were subsequently grouped (and a subset renamed) based on their sequence similarity (reviewed in Dutta and Bell 1997). Evidence that this complex was the *S. cerevisiae* replicative helicase came from three sources. First, Mcm proteins were found to move with the replication fork *in vivo* (Aparicio *et al.* 1997). Second, mutations in the Mcm2-7 complex eliminated replication-fork movement (Labib *et al.* 2000). Finally, the purified Mcm2-7 complex shows weak but detectable helicase activity (Bochman and Schwacha 2008) that is stimulated by two helicase-activating proteins (Ilves *et al.* 2010; Georgescu *et al.* 2014) that are also required *in vivo* for fork progression (Tercero *et al.* 2000; Kanemaki *et al.* 2003).

Like other replicative DNA helicases, the six Mcm2-7 subunits form a toroid with a central channel that encircles DNA. Loaded Mcm2-7 complexes are found at all origins during G_1_ phase (Wyrick *et al.* 2001). Loaded helicase cores are in the form of inactive head-to-head double hexamers of Mcm2-7 that encircle double-stranded DNA (dsDNA) (Evrin *et al.* 2009; Remus *et al.* 2009). Importantly, this opposing orientation of the Mcm2-7 rings within the double hexamer anticipates the establishment of bidirectional replication forks and suggests mechanisms for initial unwinding (see below).

**Figure 2:**
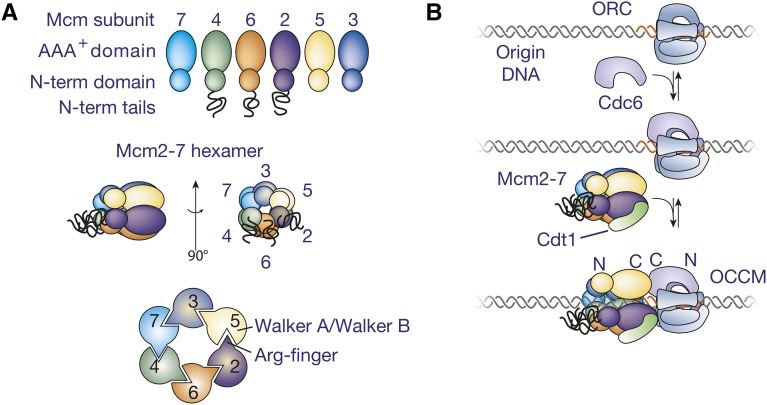
Initial recruitment of Mcm2-7 to origin DNA. (A) The six Mcm subunits share a common structure and are arranged in a ring with a defined order of the subunits. (B) A model for the events during initial recruitment of the Mcm2-7 hexamer to origin DNA. The initial ORC-Cdc6 complex is proposed to form a second ring-shaped complex of AAA+-related subunits that encircle origin DNA. This complex is proposed to recruit one Mcm2-7/Cdt1 to the adjacent DNA to form the OCCM complex. The relative position of the N- and C-terminal domains of ORC/Cdc6 and Mcm2-7 in the OCCM are labeled.

The Mcm subunits are arranged in a defined order around the ring: Mcm5-Mcm3-Mcm7-Mcm4-Mcm6-Mcm2 (Figure 2A; Davey *et al.* 2003). A high-resolution electron microscopy (EM) structure of the yeast Mcm2-7 double hexamer shows the C-terminal half of each Mcm protein contains a conserved AAA+ domain that includes Mcm-specific insertions that form β-hairpins (Li *et al.* 2015) and are predicted to interact with single-stranded DNA (ssDNA) during DNA translocation (reviewed in Bochman and Schwacha 2009). These domains form an ATPase motif at the interface between each pair of subunits and there is evidence that the six ATPases contribute differently to helicase loading, helicase activation, and DNA unwinding (Ilves *et al.* 2010; Coster *et al.* 2014; Kang *et al.* 2014). The N-terminal half of each Mcm2-7 protein can be divided into three smaller domains (Li *et al.* 2015): N-terminal subdomain A is not related to any known structure and is involved in intersubunit interactions, N-terminal subdomain B is comprised of zinc-finger motifs that mediate interactions between the two hexamers in the Mcm2-7 double hexamer (Fletcher *et al.* 2003; Fletcher *et al.* 2005; Evrin *et al.* 2014; Li *et al.* 2015), and N-terminal subdomain C is an OB-fold (OB = oligonucleotide/oligosaccharide binding) (Li *et al.* 2015) that binds ssDNA (Froelich *et al.* 2014). Although not resolved in the high-resolution structure, each Mcm2-7 protein has characteristic N- and C-terminal extensions, with the N-terminal extensions of Mcm2, Mcm4, and Mcm6 being particularly extensive (Bochman and Schwacha 2009).

In addition to ORC, Mcm2-7 loading requires two other proteins: Cdc6 and Cdt1. Cdc6 is an AAA^+^ protein in the same initiator clade as the Orc1-5 subunits and the *Escherichia coli* initiator protein DnaA (Iyer *et al.* 2004). The C-terminal portion of Cdc6 folds into a winged-helix domain (Liu *et al.* 2000), a protein fold frequently involved in DNA binding. Although ORC and Cdc6 are well conserved in other eukaryotes, *S. cerevisiae* Cdt1 is more divergent from its homologs in other eukaryotes (Devault *et al.* 2002; Tanaka and Diffley 2002), and the gene encoding budding yeast Cdt1 was originally identified by genetic interactions with topoisomerase or Sic1 (Fiorani and Bjornsti 2000; Jacobson *et al.* 2001). Despite the divergence in primary sequence, Cdt1 orthologs share a common function and are predicted to contain two winged-helix domains (Khayrutdinov *et al.* 2009).

### Helicase recruitment

The first step in helicase loading is the formation of a complex between the helicase-loading proteins and Mcm2-7 at the origin (Figure 2B), the ORC-Cdc6-Cdt1-Mcm or OCCM complex (Sun *et al.* 2013). Although normally short-lived (Ticau *et al.* 2015), inhibiting ATP hydrolysis during *in vitro* helicase-loading reactions stabilizes this complex (Randell *et al.* 2006). Only the Mcm2-7 ATPases are required to move beyond this step, although Cdc6 ATP hydrolysis also contributes (Coster *et al.* 2014; Kang *et al.* 2014).

ORC is bound to *S. cerevisiae* origins throughout the cell cycle, but the remaining proteins are only recruited as cells enter G_1_ phase (Figure 2B; Remus and Diffley 2009). Biochemical studies support a model in which ORC first interacts with Cdc6 and this complex then recruits Cdt1 and Mcm2-7 (Randell *et al.* 2006; Remus *et al.* 2009). In budding yeast, Mcm2-7 and Cdt1 are recruited to the origin as a complex (Tanaka and Diffley 2002; Remus *et al.* 2009). The C-terminal winged-helix domain of Cdt1 binds to the C-terminal region of Mcm6 (Takara and Bell 2011; Liu *et al.* 2012; Fernandez-Cid *et al.* 2013). Nuclear import of Cdt1 and Mcm2-7 is interdependent (Tanaka and Diffley 2002) and mutations that interfere with the Cdt1/Mcm6 interaction show defects in Mcm2-7 nuclear import and retention (Wu *et al.* 2012). EM and biochemical studies suggest Cdt1 also interacts with additional Mcm subunits (Fernandez-Cid *et al.* 2013; Sun *et al.* 2013).

Mcm3, Cdc6, Orc6, and Cdt1 have all been implicated in the initial recruitment of Cdt1/Mcm2-7 to the DNA-bound ORC/Cdc6 complex. Mutations in the C-terminal of Mcm3 strongly inhibit Cdt1/Mcm2-7 recruitment (Frigola *et al.* 2013; Sun *et al.* 2013). Intriguingly, Mcm2-7 recruitment requires both ORC and Cdc6, suggesting that their interaction alters the conformation of one or both proteins. The association of Orc6 with Cdt1 has also been implicated in helicase recruitment. Elimination of Orc6 prevents Cdt1/Mcm2-7 recruitment in extract-based helicase-loading experiments, and direct interactions between Orc6 and Cdt1 have been observed (Chen *et al.* 2007). In contrast, reconstituted helicase loading using purified proteins did not observe a role for Orc6 in OCCM formation (Fernandez-Cid *et al.* 2013; Frigola *et al.* 2013). Despite this discrepancy, both types of experiments agree that Orc6 is required for helicase loading.

EM studies of the OCCM complex suggest a means by which ORC/Cdc6 direct Mcm2-7 to encircle the origin DNA (Sun *et al.* 2013). In the structure, Mcm2-7 and Orc1-5/Cdc6 each form toroidal AAA+ hexamers with a shared central channel that includes additional density, which is likely to be DNA (Figure 2B). This juxtaposition suggests that binding to ORC/Cdc6 directs Mcm2-7 to encircle the adjacent DNA. Within this structure, the C-terminal AAA+ domains of Mcm2-7 interact with ORC/Cdc6. Comparison of the EM structure with a crystal structure of *Drosophila* ORC indicates that the winged-helix domains that form the C-terminal face of ORC/Cdc6 interact with Mcm2-7 (Bleichert *et al.* 2015). Consistent with an important interaction between Mcm3 and Cdc6, these two subunits are aligned within the structure and similar interactions are predicted to occur between ORC and other Mcm subunits in the OCCM.

## Opening and Closing the Ring: the Mechanism of Helicase Loading

After the initial recruitment of the helicase-loading factors and Mcm2-7 to the origin, loading of the Mcm2-7 complex onto origin DNA requires ATP hydrolysis and extensive remodeling of the interactions between these proteins. To form the final Mcm2-7 double hexamer, helicase loading necessarily involves the formation of strong interactions between the N-terminal domains of Mcm2-7 and closing of the Mcm2-7 ring around dsDNA. Importantly, the resulting loaded helicases are inactive for origin DNA melting and unwinding.

### The Mcm2/Mcm5 gate

The Mcm2-7 ring must be open during loading to provide access for origin DNA to enter the central DNA binding channel. Multiple studies indicate that a “gate” between the Mcm2 and Mcm5 subunits provides this access. DNA binding to ssDNA circles suggested that ATP binding at the Mcm2/5 interface closes the Mcm2-7 ring (Bochman and Schwacha 2008). EM studies of *Drosophila* Mcm2-7 show a gap between these subunits (Costa *et al.* 2011, 2014). Finally, artificially linking Mcm2 and Mcm5 (but not other pairs of adjacent Mcm subunits), prevents Mcm2-7 loading (Samel *et al.* 2014).

**Figure 3:**
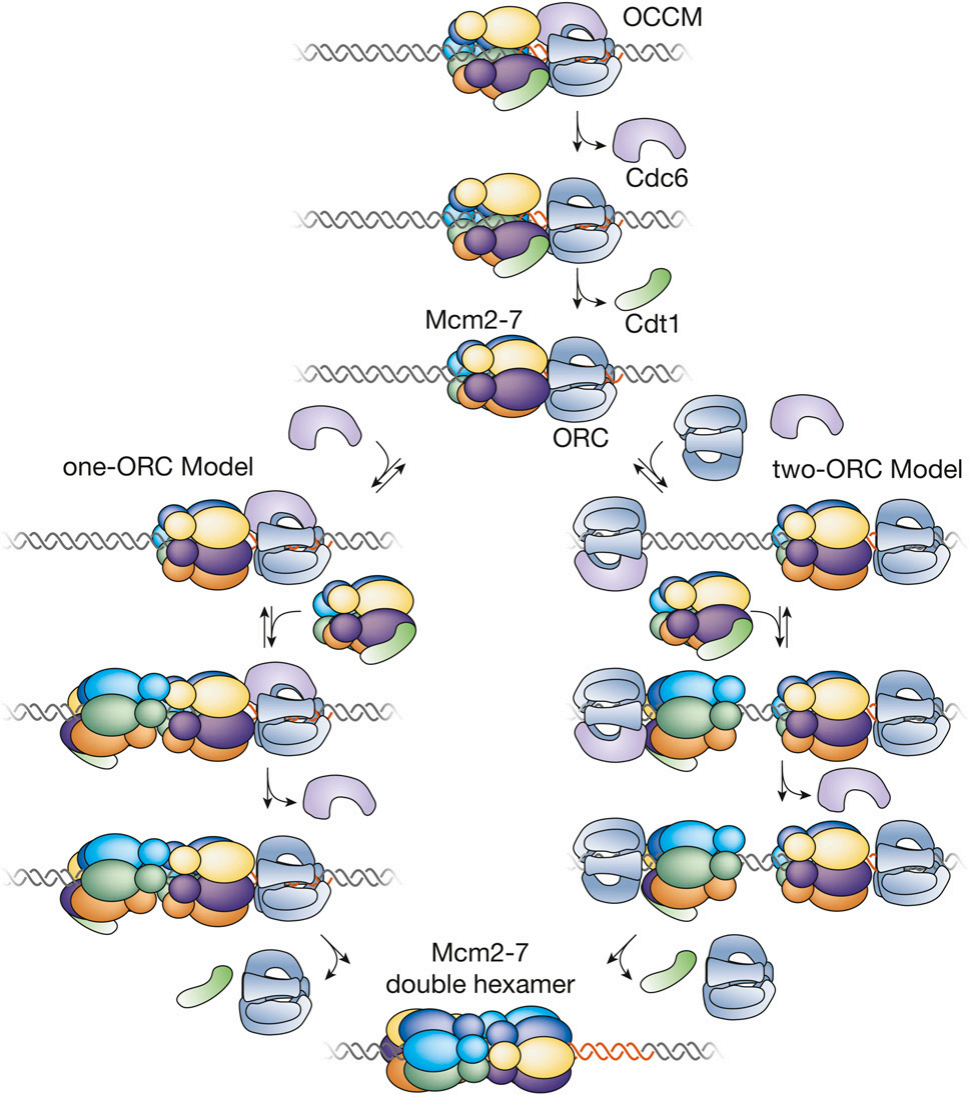
Events of helicase loading after recruitment of the Mcm2-7 complex. Two models for the events of helicase loading after formation of the OCCM are shown. The one-ORC model is based on single-molecule studies of helicase loading and predicts that the second Mcm2-7 is recruited by interactions with first Mcm2-7. The two-ORC model is based on studies suggesting the first and second Mcm2-7 is loaded by the same mechanism as the first Mcm2-7. In this model, the time of binding of the second ORC and release of the first ORC is unclear. The color code for the Mcm subunits is the same as in Figure 2A.

Once the Mcm2-7 ring has been placed around origin DNA, the ring must be sealed and maintained in a closed state to prevent release of Mcm2-7 from the origin until helicase activation. Ring closure is presumably accompanied by changes in the protein associations involved in the initial opening of the Mcm2-7 ring. Indeed, single-molecule studies show that an ordered release of Cdc6 and then Cdt1 from the OCCM (Ticau *et al.* 2015) leads to loading of each Mcm2-7 (Figure 3). If ATP binding closes the Mcm2/5 gate, it is likely that ring closure is accompanied by the prevention of ATP hydrolysis at the Mcm2/5 gate. Consistent with this hypothesis, the Mcm2-7 complex is inactive as a helicase/DNA translocase after loading. This inactivity may be due to the twisting of the N-terminal domains of each Mcm2-7 subunit with respect to the C-terminal domain in the loaded double hexamer (Sun *et al.* 2014). In contrast, these two rings are aligned vertically in the ATPase active form of the replicative helicase (Costa *et al.* 2011).

### Loading the second Mcm2-7

The head-to-head nature of the loaded Mcm2-7 double hexamer means that the two hexamers have to be loaded onto origin DNA in opposing orientations. A combination of EM and single-molecule studies has provided important insights into this process (Figure 3). The EM structure of the OCCM contains a single Mcm2-7 ring (Sun *et al.* 2013) and single-molecule studies (Ticau *et al.* 2015) indicate that each Mcm2-7 complex is recruited and loaded individually (Ticau *et al.* 2015), rather than double-hexamer formation being required for loading of Mcm2-7 around origin DNA. Each round of Mcm2-7 loading involves the ordered association and dissociation of distinct Cdc6 and Cdt1 molecules. Unlike Cdc6 and Cdt1, single-molecule studies find that one ORC molecule directs both rounds of Mcm2-7 loading during double-hexamer formation. ORC is retained after the first Mcm2-7 loading event but rapidly released after the second Mcm2-7 is loaded. These observations argue against models in which two ORC molecules bound in opposite orientations direct Mcm2-7 double-hexamer formation (Figure 3, one-ORC model). Instead, single-molecule fluorescence-energy-transfer studies support a model in which the second Mcm2-7 is recruited to the DNA through interactions with the initial Mcm2-7 complex (rather than with ORC) (Ticau *et al.* 2015). Consistent with this model, EM analysis of helicase-loading intermediates has identified a complex containing one ORC bound to a head-to-head Mcm2-7 double hexamer (Sun *et al.* 2014).

Nevertheless, other evidence supports a two-ORC model for helicase loading (Figure 3, two-ORC model). C-terminal mutations in Mcm3 that prevent binding to ORC-Cdc6 inhibit both the first and second Mcm2-7 loading event (Frigola *et al.* 2013). If only N-terminal interactions were required for recruiting the second Mcm2-7, the Mcm3 C-terminal mutant would be competent to participate as the second Mcm2-7. This suggests that the same Mcm-ORC/Cdc6 interactions are involved in the first and second Mcm2-7. In addition, although kinetically different, the similar set of protein interactions that occur during loading of the first and second Mcm2-7 are also consistent with this view (Ticau *et al.* 2015). Finally, the similarity of the B2 element to the ACS (Wilmes and Bell 2002; Liachko *et al.* 2010) could facilitate the binding of a second ORC in the opposite orientation but such a site is not present at all origins (Chang *et al.* 2011). It remains to be established whether just one of these models applies to all origins, or whether both mechanisms can function; perhaps with different origins using different mechanisms.

### Structure of the Mcm2-7 double hexamer

Unlike their archaeal orthologs that form double hexamers in solution (Brewster and Chen 2010), in yeast Mcm2-7 double hexamers are only observed after origin loading. The structure of the loaded Mcm2-7 helicases provides insights into the events of helicase loading. Cryo-EM studies of the Mcm2-7 double hexamer show that the Mcm2/5 gates of the two hexamers are not aligned (Costa *et al.* 2014; Sun *et al.* 2014; Li *et al.* 2015). Because concerted loading of two hexamers would require alignment of their Mcm2-5 gates, the two Mcm2-7 complexes in the double hexamer must be loaded sequentially. This offset structure also has the advantage of maintaining the double hexamer on DNA even if one or both Mcm2/5 gates are opened (*e.g.*, during helicase activation, see below).

Extensive interactions hold the two hexamers together (Li *et al.* 2015). Conserved zinc-finger domains found in the Mcm subunit N-termini form many interactions between the hexamers, and mutants predicted to interfere with these contacts are defective for helicase loading (Evrin *et al.* 2014). These interactions include both end-on and side-by-side associations, contributing to a 14° tilt between the two hexamer axes. Numerous Mcm subunit-specific insertions also contribute to double-hexamer formation (Li *et al.* 2015). DNA is not required to maintain the double hexamer, as these complexes are stable after extensive nuclease treatment that leaves undetectable DNA association (Evrin *et al.* 2009). Thus, ORC, Cdc6, and Cdt1 must change Mcm2-7 in a manner that facilitates double-hexamer interactions. The nature of these changes and how they are achieved is an important open question.

### Role of ATP during helicase loading

ATP binding and hydrolysis are critical for helicase loading. Indeed, 12 of the 14 proteins/subunits involved in helicase loading are related to the AAA+ family of ATPases (all but Cdt1 and Orc6) and 8 are known to bind and hydrolyze ATP (all six Mcm2-7 subunits, Orc1, and Cdc6). As described above, ATP binding by ORC and Cdc6 is required for the initial recruitment of these proteins and the Cdt1/Mcm2-7 complex to the origin. In contrast, ATP hydrolysis is required to complete Mcm2-7 loading. Mutant analysis shows that ATP hydrolysis by Mcm2-7 drives helicase loading (Coster *et al.* 2014; Kang *et al.* 2014). Cdc6 ATP hydrolysis is not required for helicase loading at high Cdc6 concentrations (Coster *et al.* 2014; Kang *et al.* 2014), but becomes important when Cdc6 concentrations are lower (Randell *et al.* 2006; Evrin *et al.* 2013; Kang *et al.* 2014). Instead, Cdc6 ATP hydrolysis is required for release of Cdc6 (under all conditions) and the release of incorrectly loaded Mcm2-7 from origin DNA (Frigola *et al.* 2013; Coster *et al.* 2014; Kang *et al.* 2014). A lack of Cdc6 release also impedes subsequent steps in helicase activation (Chang *et al.* 2015). ORC ATP hydrolysis is also not required for loading an individual Mcm2-7 double hexamer (Bowers *et al.* 2004; Evrin *et al.* 2013; Coster *et al.* 2014), but is thought to be involved in loading multiple Mcm2-7 double hexamers (Bowers *et al.* 2004; Randell *et al.* 2006).

What remains unclear is the direct consequence of ATP hydrolysis on helicase loading. As discussed above, ATP hydrolysis at the Mcm2/5 interface could influence ring opening. It is also possible that ATP hydrolysis coordinates protein dissociation events, as is seen for many ATP-controlled events. In support of this hypothesis, mutants in the Cdc6 and Mcm2-7 ATPase activity interfere with Cdt1 release from the DNA (Coster *et al.* 2014; Kang *et al.* 2014). Interestingly, the extent of the loading defect varies depending on the type of ATPase site mutant (*i.e.*, Walker A *vs.* Walker B) and the subunit that is mutated, suggesting that different Mcm ATPases regulate different events in loading (Coster *et al.* 2014; Kang *et al.* 2014).

## Few are Chosen: Helicase Activation

Helicase activation is the commitment step of replication initiation. Although loaded helicases mark all potential origins, only a subset of these sites will be used in any given cell cycle. The association and action of helicase-activating proteins selects the origins that initiate during a given cell cycle (Mantiero *et al.* 2011; S. Tanaka *et al.* 2011).

**Figure 4:**
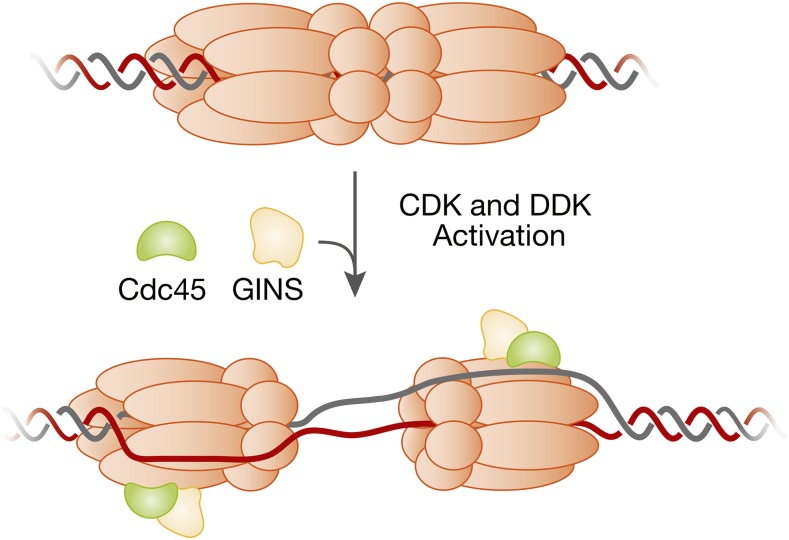
Remodeling of the Mcm2-7 double hexamer and origin DNA during helicase activation. The loaded Mcm2-7 double hexamer encircles double-stranded origin DNA (top). In contrast, the active helicase (the Cdc45/Mcm2-7/GINS or CMG complex) contains one copy of the Mcm2-7 complex and encircles ssDNA (bottom). This transition requires dissolution of the interactions between the two Mcm2-7 hexamers, melting of the origin DNA, opening of each Mcm2-7 ring, extrusion of opposite ssDNAs from the two Mcm2-7 complexes, and reclosing of the Mcm2-7 rings. The relative order of these events during helicase activation is currently unknown.

Helicase activation is more complex than helicase loading. Studies of DNA replication in *Xenopus* egg extracts indicate that activated Mcm2-7 helicases function as single hexamers encircling ssDNA (Yardimci *et al.* 2010; Fu *et al.* 2011), even though sister replication forks remain closely associated with each other in yeast cells (Kitamura *et al.* 2006). Thus, helicase activation must dramatically remodel the initially-loaded helicase and the associated DNA. The interface between the two loaded Mcm2-7 complexes must be broken and one strand of DNA expelled from each helicase, allowing the remaining DNA strand (the leading-strand template) to direct translocation (Figure 4). Triggering these events requires two kinases: the Dbf4-dependent kinase, DDK (Cdc7 kinase and Dbf4 regulatory subunit); and the cyclin-dependent kinase, S-CDK (Cdc28/Cdk1 kinase and the cyclin regulatory subunits Clb5 or Clb6). Phosphorylation of at least four proteins drives the origin association of many proteins with the loaded Mcm2-7 complex, most notably, Cdc45 (Aparicio *et al.* 1997; Zou and Stillman 1998; Tercero *et al.* 2000) and GINS (Kanemaki *et al.* 2003; Takayama *et al.* 2003). These two factors are tightly associated with Mcm2-7 at replication forks in a mutually-dependent fashion to form the activated helicase known as the Cdc45/Mcm2-7/GINS (CMG) complex (Gambus *et al.* 2006; Moyer *et al.* 2006). Helicase activation has been reconstituted with purified proteins (Yeeles *et al.* 2015), showing that all the essential factors have been identified.

### Assembling the CMG helicase

The Mcm2-7 complex is the engine of the replicative helicase but on its own it is a poor helicase (Bochman and Schwacha 2008). Association with Cdc45 and GINS dramatically stimulates the Mcm2-7 helicase (Ilves *et al.* 2010), and both Cdc45 and GINS proteins are present at replication forks and are continuously required for fork progression (Aparicio *et al.* 1997; Tercero *et al.* 2000; Kanemaki *et al.* 2003; Kanemaki and Labib 2006).

The mechanism of Mcm2-7 helicase activation by Cdc45 and GINS is still being unraveled. One possibility is that these proteins act as processivity factors for Mcm2-7, preventing release of the encircled ssDNA when the Mcm2/5 gate opens. Cdc45 and GINS form a bridge across the Mcm2/5 gate (Costa *et al.* 2011, 2014) and recent structural studies suggest that the AAA+ C-terminal domain opens the Mcm2-5 gate during DNA translocation (Abid Ali *et al.* 2016; Yuan *et al.* 2016). Cdc45 also binds ssDNA and it has been proposed that Cdc45 interacts with released ssDNA in a manner that regulates Mcm2-7 activity (Bruck and Kaplan 2013; Costa *et al.* 2014). Although this is likely to be part of the story, Cdc45 and GINS also stimulate the ATPase activity of Mcm2-7 in the absence of DNA (Ilves *et al.* 2010), indicating more direct mechanisms of stimulation also exist.

### DDK phosphorylation of Mcm2-7 drives Cdc45 recruitment

The first step in helicase activation is DDK phosphorylation of loaded Mcm2-7 complexes. The only essential target of DDK is the Mcm2-7 complex as Mcm subunit mutations bypass DDK function (Hardy *et al.* 1997; Randell *et al.* 2010; Sheu and Stillman 2010). DDK phosphorylation of the long unstructured tails of Mcm4 and Mcm6 is important for replication initiation (Randell *et al.* 2010; Sheu and Stillman 2010). Many of these Mcm4 and Mcm6 DDK phosphorylation sites require prior (or priming) phosphorylation of Mcm2-7 by Mec1 and/or CDK (Francis *et al.* 2009; Randell *et al.* 2010). DDK binds Mcm2-7 and regions within the Mcm4 and Mcm2 N-terminal tails mediate this interaction (Sheu and Stillman 2006; Francis *et al.* 2009). Both DDK phosphorylation of, and binding to, Mcm2-7 is stimulated by double-hexamer formation (Francis *et al.* 2009; Sun *et al.* 2014); perhaps due to Cdc7 and Dbf4 binding different Mcm subunits that are only in close proximity in the context of the double hexamer (Ramer *et al.* 2013; Sun *et al.* 2014).

**Figure 5:**
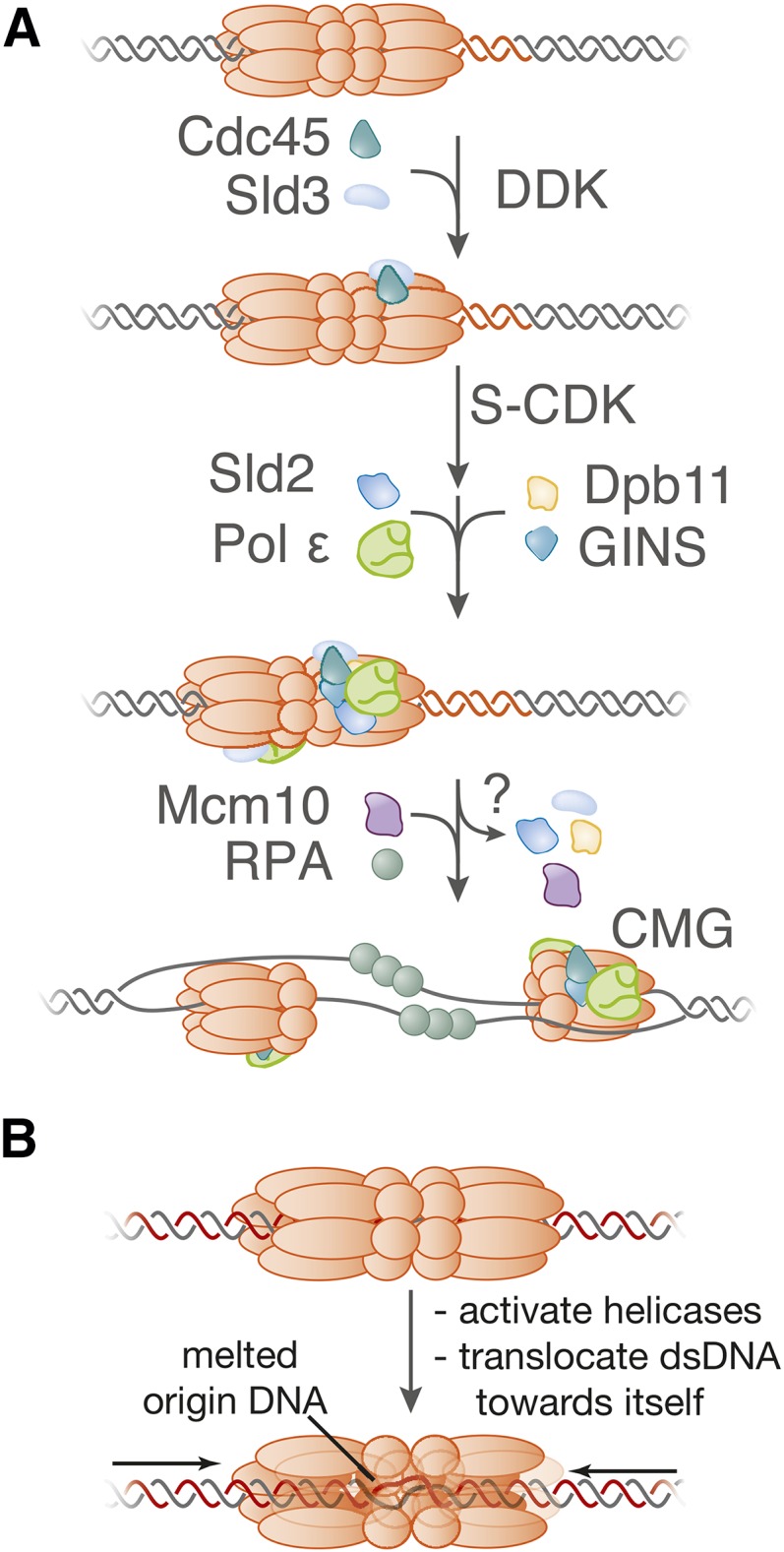
A model for helicase activation during the initiation of DNA replication. (A) The model illustrates the first time that each factor is required. Although Sld2, Sld3, and Dpb11 are not thought to be part of the final replisome; it is unclear when these factors are released. Helicase activation is associated with the recruitment of many additional factors to form the replisome (see below). (B) A model for the mechanism of initial origin DNA melting by the Mcm2-7 double hexamer.

DDK phosphorylation drives recruitment of Cdc45 and Sld3 to the Mcm2-7 double hexamer (Figure 5A). *In vivo*, recruitment of Cdc45 and Sld3 to origins is interdependent (Kamimura *et al.* 2001; Kanemaki and Labib 2006; Heller *et al.* 2011), but Sld3 can be recruited to loaded Mcm2-7 complexes without Cdc45 *in vitro* (Deegan *et al.* 2016). Sld3 binds to phosphorylated peptides in Mcm4 and Mcm6, indicating that Sld3 recruits Cdc45 to the phosphorylated Mcm2-7 double hexamer. Although nonessential for replication (T. Tanaka *et al.* 2011; Deegan *et al.* 2016), Sld7 binds and stabilizes Sld3 and associates with origin DNA in an Sld3-dependent manner (T. Tanaka *et al.* 2011). Intriguingly, deletion of part of the Mcm4 N-terminal extension bypasses DDK function (Sheu and Stillman 2010), suggesting that DDK phosphorylation relieves inhibition caused by this region of Mcm4, perhaps by revealing a binding site(s) for Sld3.

The initial events of CMG formation are observed in G_1_-phase cells. Cdc45, Sld3 and Sld7 each associate with early-initiating origins of replication during G_1_ phase (Aparicio *et al.* 1999; Kanemaki and Labib 2006; T. Tanaka *et al.* 2011). Although DDK is downregulated during G_1_ (Cheng *et al.* 1999; Weinreich and Stillman 1999; Godinho Ferreira *et al.* 2000), the association of Cdc45-Sld3-Sld7 with origins in G_1_ is dependent upon DDK activity but independent of S-CDK activity (Heller *et al.* 2011; S. Tanaka *et al.* 2011).

### CDK phosphorylation of Sld2 and Sld3 drives recruitment of GINS to origins

The recruitment of GINS and the completion of CMG-complex formation require S-CDK activity (Figure 5A). There are two essential CDK targets during replication initiation: Sld2 and Sld3 (Tanaka *et al.* 2007; Zegerman and Diffley 2007; Yeeles *et al.* 2015). Phosphorylation of Sld2 and Sld3 leads each protein to bind different pairs of BRCT (BRCA1 C-Terminus) repeats in Dpb11 that act as phosphorylation-dependent binding domains. The CDK-dependent interaction between Sld2 and Dpb11 stimulates interactions of these proteins with GINS and DNA polymerase (Pol) ε to form the preloading complex (pre-LC), which is labile but can be detected during S phase (Muramatsu *et al.* 2010). The phosphorylation-dependent interaction between Sld3 and the pre-LC-associated Dpb11 recruits the latter to the origin, via Sld3-bound Mcm2-7. Consistent with this model, mutations that bypass the phosphorylation-dependent interactions of Sld2-Dpb11-Sld3 result in S-CDK-independent DNA replication (Tanaka *et al.* 2007; Zegerman and Diffley 2007). Despite this, replication under such conditions is inefficient, indicating either that the suppressor mutations are not fully effective or that other CDK targets (*e.g.*, Mcm2-7) also contribute to the initiation of chromosome replication.

Additional interactions are important for CMG formation. Two-hybrid interactions between GINS, Cdc45, and Sld3 have been detected and structural studies support direct interactions between Cdc45 and GINS (Costa *et al.* 2011; Abid Ali *et al.* 2016; Yuan *et al.* 2016). These interactions are likely responsible for the increased origin association of Cdc45 that is observed when yeast cells enter S phase (Zou and Stillman 1998; Aparicio *et al.* 1999; Kanemaki and Labib 2006). In addition, the region between the two pairs of BRCT repeats in Dpb11 binds GINS and is also required for CMG formation (Tanaka *et al.* 2013). Similarly, a critical interaction between the second subunit of DNA Pol ε (Dpb2) and a GINS subunit is required for CMG assembly (Sengupta *et al.* 2013). Therefore, DNA Pol ε plays an essential role in the initiation of chromosome duplication, even before synthesis of any DNA.

### Activation of DNA unwinding

Studies of Mcm10 suggest that CMG-complex formation is not sufficient to initiate DNA unwinding at the origin (Figure 5A). Elimination of Mcm10 function does not block recruitment of Cdc45 and GINS to origins, but instead prevents binding of the eukaryotic ssDNA binding protein, RPA, to origin-proximal DNA (van Deursen *et al.* 2012; Watase *et al.* 2012). Mcm10 associates preferentially with the loaded double hexamer of Mcm2-7 (van Deursen *et al.* 2012) and has been detected at origins even during G_1_ phase (Ricke and Bielinsky 2004). Once cells enter S phase, however, Mcm10 accumulates at origins in a manner requiring CDK activity and initial CMG assembly but independent of origin unwinding (Heller *et al.* 2011; van Deursen *et al.* 2012; Watase *et al.* 2012). Together, these studies suggest that Mcm10 activates the CMG complex, stimulating DNA unwinding and RPA binding to the resulting ssDNA. This function could explain why Mcm10 is required for DNA Pol α recruitment to the origin (Ricke and Bielinsky 2004; Heller *et al.* 2011), because DNA Pol α recruitment depends on origin unwinding (Heller *et al.* 2011) and DNA Pol α binds RPA (Dornreiter *et al.* 1992). The mechanism of Mcm10 activation is unknown but could include facilitating separation of the two Mcm2-7 hexamers (Quan *et al.* 2015), ssDNA extrusion from Mcm2-7, or DNA melting.

### Remodeling at the origin

The isolated CMG complex contains one Mcm2-7 complex (Gambus *et al.* 2006) and current data indicate that a single CMG helicase moves in a 3′ to 5′ direction on ssDNA at each fork (Yardimci *et al.* 2010; Fu *et al.* 2011; Sun *et al.* 2015). If so, there must be significant remodeling of the initially-loaded helicases and their associated DNA during initiation (Figure 4): (1) the interactions between the two Mcm2-7 complexes in the initial double hexamer must be broken; (2) the origin DNA must be melted; and (3) the lagging-strand template must be excluded from each of the Mcm2-7 complexes central channel, which requires the opening and closing of the Mcm2-7 ring. The order of these events and what proteins drive them remain largely unknown, however, Mcm2-7 (see below), Cdc45, GINS, and Mcm10 (see above) represent possible candidates.

One insight into the strand exclusion process comes from a recent crystal structure of the N-terminal domain of the archaeal homohexameric MCM bound to ssDNA (Froelich *et al.* 2014). These studies found ssDNA bound to the MCM-ring interior, perpendicular to the central channel with a defined polarity. Intriguingly, the polarity of the MCM ssDNA binding domain (MSSB) predicts that upon melting of the origin DNA, the MSSB would capture the ssDNA that will become the CMG-translocating strand (the leading-strand DNA template). Importantly, Mcm2-7 mutations predicted to interfere with these interactions exhibit defects in helicase activation.

How does the initial unwinding of origin DNA occur? One intriguing possibility is that origin DNA melting is driven by activation of the CMG helicase before separation of the double hexamer (Figure 5B). Based on the polarity of the CMG helicase, activation in the context of the double hexamer would pump dsDNA in the central channel toward the double-hexamer interface, straining the interactions between strands. Structural studies of the Mcm2-7 double hexamer reveal a kink in the central channel (near the MSSB) that would deform dsDNA, potentially acting as a nucleation site of DNA unwinding (Li *et al.* 2015). This model demands that initial DNA melting anticipates double-hexamer separation and is further supported by the observation that MCM helicases can translocate dsDNA (Kaplan and O’Donnell 2004).

## When to Begin: Temporal Control of Origin Activation

There are two properties of an origin of replication that can be measured within a population of cells: the average time within S phase that an origin initiates (origin timing) and the percentage of cell divisions that a particular origin initiates (origin efficiency). These two properties are connected because the earlier an origin initiates, the less likely it will be inactivated by the passage of a replication fork derived from an adjacent origin. Thus, origins that initiate early in S phase (early-firing origins) tend to be more efficient than those that fire later in S phase. This distribution of replication origin firing across S phase is observed in most eukaryotic cells (the primary exceptions being some early embryonic cells).

The most likely reason for distributing the time of origin activation across S phase is to ensure complete genome replication. The regulation of eukaryotic DNA replication (see below) prevents reloading of the Mcm2-7 helicase core (except in rare instances, see Lydeard *et al.* 2010). Thus, if all replication origins initiated simultaneously upon S-phase entry, there would be no way to complete duplication of the intervening DNA if two converging replication forks both stalled or collapsed. By reserving a subset of origins to initiate later in S phase, activation of an origin located between the collapsed replication forks can complete replication.

The chromosome context of an origin influences its time of replication initiation. For example, when the minimal DNA region encoding the early-firing *ARS1* and late-firing *ARS501* were substituted for one another, they each assumed the timing of the origin they replaced rather than bringing their replication time to the new locus (Ferguson and Fangman 1992). A similar analysis of a larger number of origins found that many origins showed the same chromatin dependence, however, a subset of early-initiating origins retained an early-firing time even when inserted into a late-chromatin neighborhood (Looke *et al.* 2013). Early-firing origins are enriched for origins that show enhanced ORC DNA binding (Hoggard *et al.* 2013) and Mcm2-7 loading (Das *et al.* 2015) and more frequently retain ORC binding throughout the cell cycle (Belsky *et al.* 2015). Finally, localization of the Rpd3 histone deacetylase near an origin delays initiation, and deletion of Rpd3 leads to earlier firing of a subset of origins (Vogelauer *et al.* 2002; Knott *et al.* 2009).

Two chromosome landmarks have consistent effects on replication timing: centromeres and telomeres. Origins proximal to centromeres are among the earliest replicating (Raghuraman *et al.* 2001) and this property requires an active centromere. Eliminating centromere function eliminates the early firing of adjacent origins and insertion of an active centromere proximal to an origin makes it early firing (Pohl *et al.* 2012). Telomere proximity has the opposite effect on replication timing, with origins within 35 kb of telomeres typically initiating late in S phase (Raghuraman *et al.* 2001). The size of the telomere influences this effect. Telomeres of normal length delay initiation of proximal origins, whereas short telomeres result in early replication of telomere-proximal origins (Bianchi and Shore 2007).

### Program or probability? Control of replication timing

One can consider two extreme models for the control of replication timing. One possibility is that replication origins follow a predetermined order with each origin initiating at a defined time in S phase. The extreme form of this type of model would be a domino model in which initiation of one origin is required for initiation of the next origin in the program. Alternatively, the time of origin initiation could be controlled stochastically, with each origin competing for limiting replication proteins. In this model, replication timing would be controlled by differing abilities of origins to compete for replication factors.

Increasing evidence has accumulated in favor of a stochastic-competition model for replication timing in yeasts (Bechhoefer and Rhind 2012; Kaykov and Nurse 2015). In contrast to a tightly-deterministic model, measurement of percent replication of any given origin shows a gradual transition from unreplicated to replicated (Ferguson *et al.* 1991). Although this distribution could be due to lack of cell synchrony, single-molecule studies of nucleotide incorporation into *S. cerevisiae* chromosome VI show stochastic origin usage (Czajkowsky *et al.* 2008). The observed patterns show different subsets of origins are used each cell cycle. Strikingly, clear examples of early-firing origins initiating after late-firing origins were among the patterns observed.

Consistent with a stochastic-competition model, changing the concentration of limiting replication factors or the number of competing origins alters replication timing. Overexpression of a subset of limiting helicase-activating proteins advances the time of replication of ordinarily late-firing origins of replication (Mantiero *et al.* 2011; S. Tanaka *et al.* 2011). These findings suggest that the helicase-activation step is rate-limiting for initiation. Similarly, changing the number of competing origins alters global replication timing (Yoshida *et al.* 2014). Either increasing or decreasing the percentage of origins that initiate affects the time of initiation of other origins in the genome.

### Chromatin factors influence replication timing

Although the evidence in favor of a stochastic-competition model is strong, the characteristics that allow some origins to compete more effectively for limiting replication factors remain unclear. There are two basic ways to envision regulating the ability to compete: (1) modulating the accessibility of the origin to the limiting replication factors, and (2) altering the local activity of a limiting replication factor. Studies of the mechanisms controlling replication timing have identified mechanisms of both types.

The late initiation of telomere-proximal origins provides an example of control by accessibility. Mutations in the Sir3 protein, a key component of telomeric heterochromatin, result in earlier replication initiation for telomere-proximal origins (Stevenson and Gottschling 1999). Sir3 is required for the formation of silent chromatin structures at telomere-repeat origins, which inhibit DNA accessibility to many proteins (Oppikofer *et al.* 2013); presumably including one or more of the limiting replication proteins.

Telomeres and centromeres regulate replication initiation time by modulating the local activity of a limiting replication protein. The early initiation of centromere-proximal origins is mediated by increasing local DDK concentration through an interaction between DDK and the Ctf19 kinetochore complex (Natsume *et al.* 2013). A mutation in Dbf4 that prevents kinetochore localization, or deletion of Ctf19, delays centromere-proximal origin initiation without altering the timing of other origins. The telomere-binding protein Rif1 acts in the opposite way; inhibiting DDK activity proximal to telomeres. Rif1 binds to both Dbf4 and a PP1 phosphatase, Glc7. Mutations that eliminate Rif1 binding to Glc7 are able to suppress DDK mutants and advance the time of initiation of telomere-proximal origins (Davé *et al.* 2014; Hiraga *et al.* 2014; Mattarocci *et al.* 2014). Interactions of Rif1 with Dbf4 are thought to help target Glc7 to sites of DDK action. In this case, it is the recruitment of the DDK-counteracting Glc7 phosphatase to the telomere that delays the local times of replication initiation.

The yeast forkhead box (Fox) transcription factors are also implicated in the control of replication timing. Binding sites for these proteins are enriched near early-initiating origins and depleted from late-initiating origins (Knott *et al.* 2012). Elimination of Fkh binding sites proximal to early origins delays their time of replication initiation (Knott *et al.* 2012), although Fkh1/2 binding proximal to an origin is not sufficient to impart early replication initiation (Knott *et al.* 2012; Looke *et al.* 2013). Mapping of interchromosomal interactions across the *S. cerevisiae* genome showed that early-initiating origins are found in two clusters, and the interaction between the early-replicating *ARS305* origin and other Fkh1/2-activated origins is impaired in Fkh1/2 mutant cells (Knott *et al.* 2012). Together, these studies support a model in which Fkh1/2 interactions facilitate clustering of early-initiating origins and that this clustering gives these origins an advantage when competing for limiting replication proteins.

Together these findings suggest a model for the control of replication origin timing by intranuclear localization. First, origins with similar or coordinated times of initiation are held together in the nucleus. Second, early-firing clusters of origins enhance the local concentration of replication initiation factors (*e.g.*, high local concentrations of DDK recruited by the kinetochore). Although centromeres recruit a limiting factor, it is unclear what allows Fkh1/2-activated origins to recruit a limiting factor(s). This model does not exclude a role for different levels of Mcm2-7 loading, local chromatin structures, and histone modification in further modulating the replication times of origins. Genome-wide studies show a correlation between early replication firing and increased Mcm2-7 loading (Das *et al.* 2015). In addition, once loaded, Mcm2-7 double hexamers are closely associated with one of the two adjacent-positioned nucleosomes (Belsky *et al.* 2015), suggesting that local nucleosome positioning and modification influences Mcm2-7 accessibility.

## Never Again: Cell-Cycle Control of Replication Initiation

**Figure 6:**
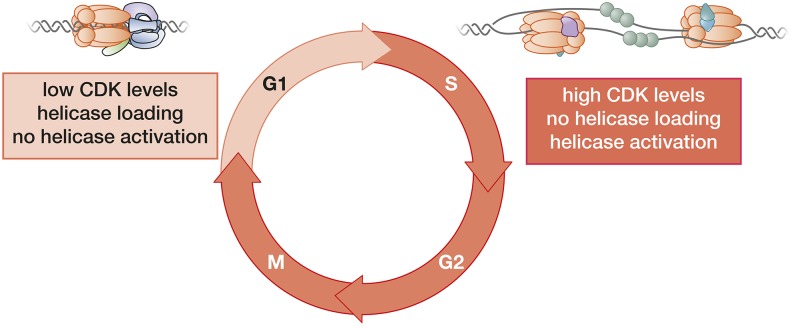
Helicase loading and activation are segregated during the cell cycle. The cell cycle can be split into two phases with respect to DNA replication. Helicase loading only occurs in G_1_ phase when CDK levels are low. The increased CDK levels present during S, G_2_, and M phases prevent helicase loading through multiple mechanisms. The same elevated CDK levels are required to activate CMG assembly and helicase activation, ensuring no helicase is activated during G_1_ phase. This regulation ensures no origin can initiate more than once per cell cycle.

It is critical that the eukaryotic genome is replicated both completely and exactly once per cell cycle. Even a few origins initiating more than once in a cell cycle can be lethal to cells or result in genome rearrangement (Green and Li 2005; Green *et al.* 2010). The primary mechanism to ensure a single round of replication per cell cycle is the temporal separation of helicase loading from helicase activation and replisome assembly (Figure 6). Throughout eukaryotic organisms this is achieved by tightly-restricting helicase loading to the G_1_ phase of the cell cycle and helicase activation to S phase (Remus and Diffley 2009). In this way, cells have only one opportunity to license their origins through helicase loading and one opportunity to activate the loaded helicases per cell cycle. This regulation is particularly well understood in *S. cerevisiae* cells, where the regulation is controlled by the cell-cycle oscillation of CDK activity.

Helicase loading is tightly restricted to the G_1_ phase of the cell cycle to ensure that no origin of replication can reload Mcm2-7 at an origin that has initiated replication (Arias and Walter 2007). At least three different mechanisms prevent helicase loading in *S. cerevisiae* cells. Each of these mechanisms is mediated by CDK phosphorylation of helicase-loading proteins. CDK phosphorylation of Cdc6 leads to its ubiquitin-mediated degradation (Drury *et al.* 2000). CDK phosphorylation of Mcm3 results in the nuclear export of Mcm2-7 proteins that are not loaded onto origin DNA (Labib *et al.* 1999; Nguyen *et al.* 2000). Phosphorylation of ORC directly interferes with helicase loading, although the mechanism of inhibition is unclear (Chen and Bell 2011; Fernandez-Cid *et al.* 2013; Frigola *et al.* 2013). Finally, an RXL or Cy motif on Orc6 recruits Clb5 (the primary S-phase cyclin) to ORC, which presumably localizes CDK action to the origin and potentially directly inhibits loading (Wilmes *et al.* 2004). Simultaneous elimination of all of these mechanisms either by mutating phosphorylation/binding sites or overriding the control mechanism results in uncontrolled replication and cell death (Nguyen *et al.* 2001). Consistent with all of the inhibitory mechanisms being mediated by CDK phosphorylation, inhibition of CDK activity outside of G_1_ leads to a new round of helicase loading and, when CDK activity is restored, rereplication of the genome (Dahmann *et al.* 1995).

For the regulation described above to prevent reinitiation there can be no time during normal cell division when both helicase loading and activation occur. Such a situation would be most likely to occur during the transition between the two states. During the G_1_- to S-phase transition, a mechanism to cleanly separate the two states arises from the finding that both G_1_ and B-type CDKs can phosphorylate Cdc6 (Drury *et al.* 2000). G_1_ CDKs become activated at the end of G_1_, triggering Cdc6 degradation after helicase loading has occurred. Because G_1_ CDKs are required for activation of the S-phase cyclins, there is a window during which neither helicase loading (due to the presence of G_1_-CDK activity) nor helicase activation (due to lack of S-CDK activity) can occur. The sequential degradation of the Dbf4 subunit of DDK (required for helicase activation) and B-type cyclins (that prevent helicase loading) at the M–G_1_ transition, ensures that helicase activation is inhibited prior to new helicase loading. Importantly, all loaded helicases are removed as a consequence of DNA replication, either due to their use in replisome assembly or their removal by replication forks generated at an adjacent origin (Santocanale and Diffley 1996).

Although it is tempting to consider the multiple mechanisms that inhibit helicase loading outside of G_1_ as redundant, this is not the case. Analyses of mutants that are defective for a subset of mechanisms show reinitiation from a specific subset of origins (Green and Li 2005). In addition, although loss of an individual mechanism is not essential in any single cell division, the full set of mechanisms is critical to maintaining genomic stability over many generations and throughout a population (Diffley 2010).

## Putting Things Together: Building the Replisome

In principle, the helicase, polymerases and many of the other factors required to duplicate eukaryotic chromosomal DNA can function in isolation from each other, yet a subset of these factors assemble with many other proteins to form the replisome at yeast DNA replication forks. Past studies of DNA replication in bacteria point to two important reasons for replisome assembly: first to allow tight coupling between DNA unwinding and DNA synthesis, thus minimizing the exposure of ssDNA; and second to allow a fast DNA polymerase to stimulate the rate of unwinding by an otherwise slow DNA helicase (Kim *et al.* 1996). The same principles apply to budding yeast, yet the eukaryotic replisome is more complex and enigmatic than its bacterial counterpart, reflecting the need to duplicate a eukaryotic chromosome in all its complexity (chromatin, epigenetics, cohesion, *etc*.), and not just facilitate efficient DNA synthesis.

### Insights into the eukaryotic DNA replication fork from studies of SV40 viral DNA replication

Much of our understanding of the yeast DNA replication machinery is founded on earlier biochemical studies of SV40 viral DNA replication in extracts of human cells. Reconstitution of SV40 replication *in vitro* facilitated the identification and mechanistic study of multiple DNA replication factors, since the only viral protein required for SV40 DNA synthesis is T-antigen, which replaces the CMG helicase at the SV40 replication fork (Kelly 1988; Hurwitz *et al.* 1990; Waga and Stillman 1998; Fanning and Zhao 2009). However, by using T-antigen, SV40 dispenses with the cellular-initiation machinery and the leading-strand DNA polymerase that is physically linked to the CMG helicase. Therefore, SV40 studies left many questions unanswered regarding the replicative helicase, the initiation mechanism, leading-strand synthesis, and the regulation of chromosome replication in eukaryotic cells.

### Genetic evidence for the division of labor at the yeast replication fork

DNA can only be synthesized in a 5′ to 3′ direction, so each fork has a leading strand that is extended continuously in the same direction as helicase progression, and a lagging strand that is made discontinuously as a series of Okazaki fragments. Moreover, the DNA polymerases at replication forks are only able to extend preexisting strands. Therefore, each new DNA molecule must be started by the synthesis and extension of a short RNA molecule.

Three multi-subunit DNA polymerases, Pol α, Pol δ, and Pol ε, are essential for DNA replication in budding yeast and each has a distinct role at replication forks (Kunkel and Burgers 2014). Only Pol α can begin new DNA strands by the concerted action of its heterodimeric primase subunits, which synthesize 8–10 nt RNAs, and the Pol1 DNA polymerase subunit, which extends each RNA primer with about 10–15 nt of DNA (Pellegrini 2012). Although Pol α is unique in its ability to make and extend RNA primers, it is ill-suited for extensive DNA replication as it has limited processivity (Perera *et al.* 2013), lacks a proofreading exonuclease and, thus, makes frequent errors. As described below, other factors normally prevent Pol α from extending the initial RNA-DNA primers at replication forks (Georgescu *et al.* 2014, 2015).

In contrast to Pol α, both Pol ε and Pol δ are capable of highly-processive DNA synthesis and include a proofreading exonuclease that greatly reduces the rate of errors during DNA synthesis. The latter feature provided an avenue to explore the division of labor between Pol ε and Pol δ at budding yeast DNA replication forks. Mutations in the exonuclease domains of Pol2 (Pol ε) or Pol3 (Pol δ) increased the rate of specific mutations in a marker gene placed close to a highly-active origin of DNA replication. By placing the marker in each of the two possible orientations relative to the origin, cells with mutated Pol2 or Pol3 showed distinct spectra of mutations. Importantly, these mutations indicated that Pol ε and Pol δ proofread errors on opposite DNA strands of the fork (Shcherbakova and Pavlov 1996). Similar experiments involving catalytic mutations in Pol2 and Pol3 that increase the rate of specific misincorporations, showed that Pol ε was almost-exclusively responsible for extending the leading strand at replication forks (Pursell *et al.* 2007), whereas Pol δ completes each Okazaki fragment on the lagging strand (Nick McElhinny *et al.* 2008). Consistent with this view, experiments with mutagenic Pol1 (catalytic subunit of Pol α) indicated that lagging-strand mutations created by Pol α are corrected by the exonuclease activity of Pol δ (Pavlov *et al.* 2006).

One caveat of these studies is that they provide a low-resolution view of DNA polymerase action, as the error rates of mutator polymerases from mismatches during DNA synthesis are on the order of 1 in 10^7^ bases synthesized. Subsequently, a much higher resolution view was obtained by monitoring ribonucleotide incorporation into DNA, in cells that lack ribonucleotide excision repair (see below, *Avoiding errors during DNA synthesis*). Ribonucleotides are incorporated at frequencies of 10^−3^ to 10^−4^ during DNA synthesis, rising to 10^−2^ to 10^−3^ in cells with mutator polymerases (Clausen *et al.* 2015; Koh *et al.* 2015). This property facilitated a genome-wide analysis of polymerase usage, confirming that Pol ε replicates the leading strand, whereas Pol α and Pol δ synthesize the lagging strand.

**Figure 7:**
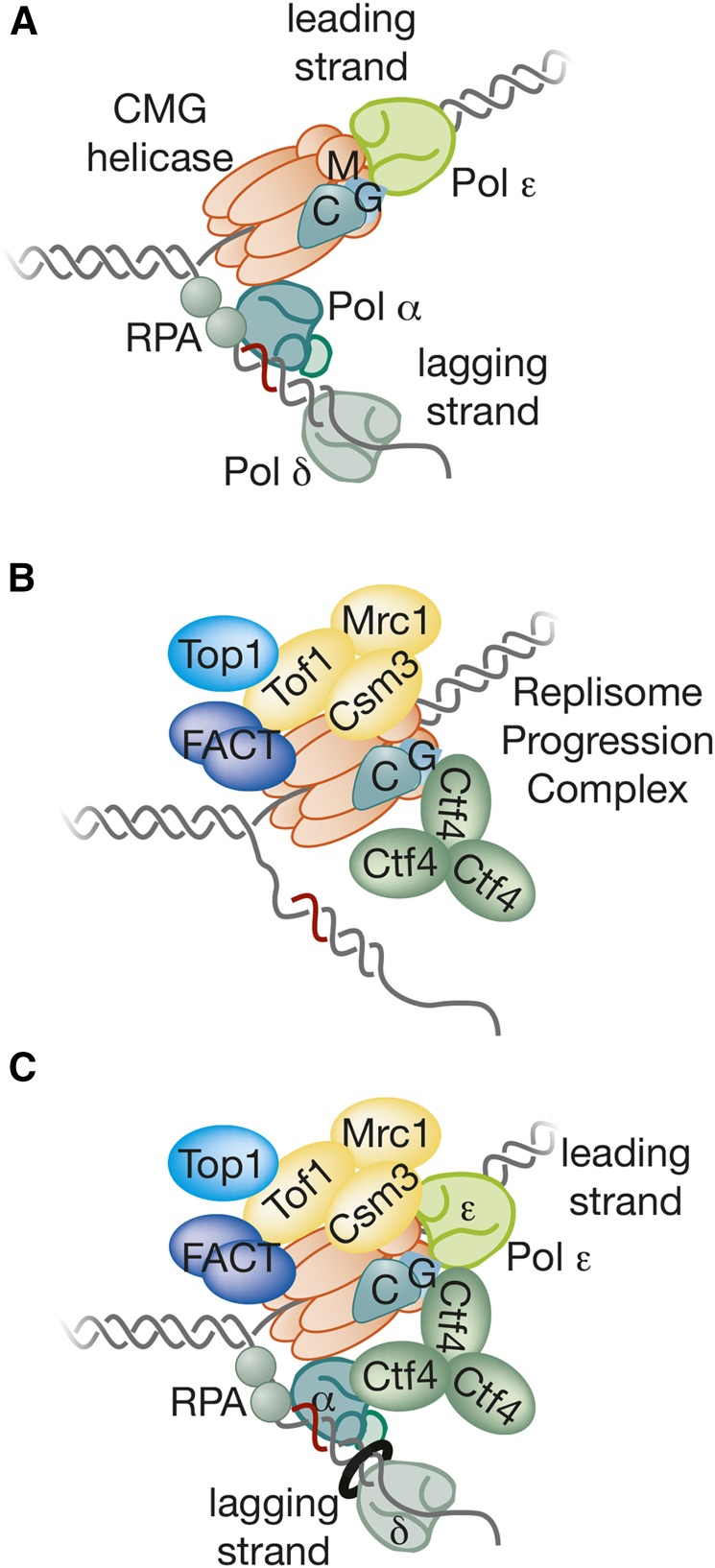
Building the replisome. (A) The division of labor among DNA polymerases at the yeast replication fork. (B) The RPC assembles around the CMG helicase at replication forks. (C) The RPC is connected to Pol ε and Pol α at forks, but apparently not to Pol δ.

It has been suggested that the mutator polymerase data could be explained by an alternative model, whereby Pol δ synthesizes both leading and lagging strands, and Pol ε only proofreads errors that are made during leading-strand synthesis (Johnson *et al.* 2015). However, it is important to note that ribonucleotide incorporation is about twofold higher for the leading strand compared to the lagging strand in cells with wild-type DNA polymerases (Clausen *et al.* 2015; Koh *et al.* 2015). This figure matches the predicted frequency of ribonucleotide incorporation for the two strands, based on *in vitro* measurements of the frequency of ribonucleotide incorporation by the three DNA polymerases and the assumption that Pol ε replicates the leading strand and Pol α or Pol δ synthesize the lagging strand (Nick McElhinny *et al.* 2010b). Moreover, Pol ε was found to associate with the leading strand and Pol δ with the lagging strand in ChIP studies combined with strand-specific DNA sequencing (Yu *et al.* 2014). Together, these findings strongly support the original division of labor in wild-type yeast cells (Figure 7A). Biochemical studies further support this view, as discussed in the following sections.

### Pol ε and Pol α are connected to the CMG helicase as part of the replisome

The Dpb2 subunit of Pol ε has a GINS-binding domain that serves two important functions during replication. First, it is essential for GINS recruitment to origins during helicase assembly (see above; Muramatsu *et al.* 2010; Sengupta *et al.* 2013), and second it is required to tether Pol ε to the CMG helicase at replication forks (Sengupta *et al.* 2013; Langston *et al.* 2014). Additional contacts between Pol ε and CMG are indicated both by EM, as well as by a combination of chemical cross-linking and mass spectrometry (Sun *et al.* 2015). Thus, the C-terminal half of Pol2 appears to contact Mcm2, Mcm6, and Cdc45, with Dpb2 being cross-linked to the C-terminal half of Mcm5 in addition to the Psf1 subunit of GINS.

Pol α is connected indirectly to the CMG helicase via Ctf4 (Zhu *et al.* 2007; Gambus *et al.* 2009; Tanaka *et al.* 2009), which forms a trimer with three identical binding sites for a short peptide motif found in GINS and Pol α (Simon *et al.* 2014). Initially, this finding suggested that Ctf4 might link one or two Pol α complexes to the CMG helicase at replication forks, analogous to the presence of two lagging-strand polymerases in the *E.coli* replisome (McInerney *et al.* 2007; Reyes-Lamothe *et al.* 2010). However, it now appears that the Ctf4 trimer uses the same binding sites to bind many proteins in addition to GINS and Pol α, suggesting that Ctf4 is a hub that connects CMG to a broader set of client proteins (Villa *et al*. 2016). The functional significance of Ctf4 coupling Pol α to the CMG helicase within the replisome remains to be explored, and Ctf4 is not limiting for priming by Pol α during *in vitro* DNA replication (Yeeles *et al.* 2015). The *in vivo* association of Pol α with CMG is greatly reduced in the absence of Ctf4 (Gambus *et al.* 2009), but is not abolished (Sengupta *et al.* 2013), and recent work suggests that direct association of Pol α with CMG supports efficient priming during lagging-strand synthesis (Georgescu *et al.* 2015).

In contrast to Pol ε and Pol α, there is no evidence that Pol δ is connected to the CMG helicase. Pol δ does not copurify with CMG under conditions that preserve the interactions of CMG with Pol ε and Ctf4-Pol α (De Piccoli *et al.* 2012; Sengupta *et al.* 2013). Thus, it seems likely that the extension of Okazaki fragments by Pol δ is uncoupled from the action of the CMG helicase, unlike synthesis of the leading strand by Pol ε.

## Recruitment and Suppression Mechanisms Establish the Division of Labor at Replication Forks

Studies of SV40 replication showed that multiple factors compete for access to the 3′ end, following the release of Pol α from an RNA-DNA primer (Kelly 1988; Waga and Stillman 1998; Hurwitz *et al.* 1990; Fanning and Zhao 2009). Similarly, budding yeast Pol α is able to extend both leading and lagging strands *in vitro* in the absence of Pol ε and Pol δ (Georgescu *et al.* 2015), but at replication forks Pol α is excluded by other factors. In the presence of the CMG helicase, yeast Pol ε supports efficient leading-strand synthesis *in vitro*, outcompeting both Pol α and Pol δ (Georgescu *et al.* 2014, 2015). It is likely that the physical association with the CMG helicase explains both the preference of Pol ε for leading-strand synthesis and the mechanism by which Pol ε suppresses both Pol α and Pol δ during leading-strand synthesis. Consistent with this view, point mutations that kill the catalytic activity of Pol ε are lethal (Dua *et al.* 1999), presumably because inactive Pol ε prevents Pol α and Pol δ from accessing the leading strand. In contrast, displacement of Pol ε from CMG after initiation (Sengupta *et al.* 2013) or deletion of the catalytic domain of Pol2 (Dua *et al.* 1999; Kesti *et al.* 1999) is not lethal but leads to very slow DNA replication and very poor growth of yeast cells (Dua *et al.* 1999; Kesti *et al.* 1999; Sengupta *et al.* 2013). Leading-strand synthesis under such conditions is likely mediated by Pol δ and/or Pol α, though this remains to be demonstrated.

**Figure 8:**
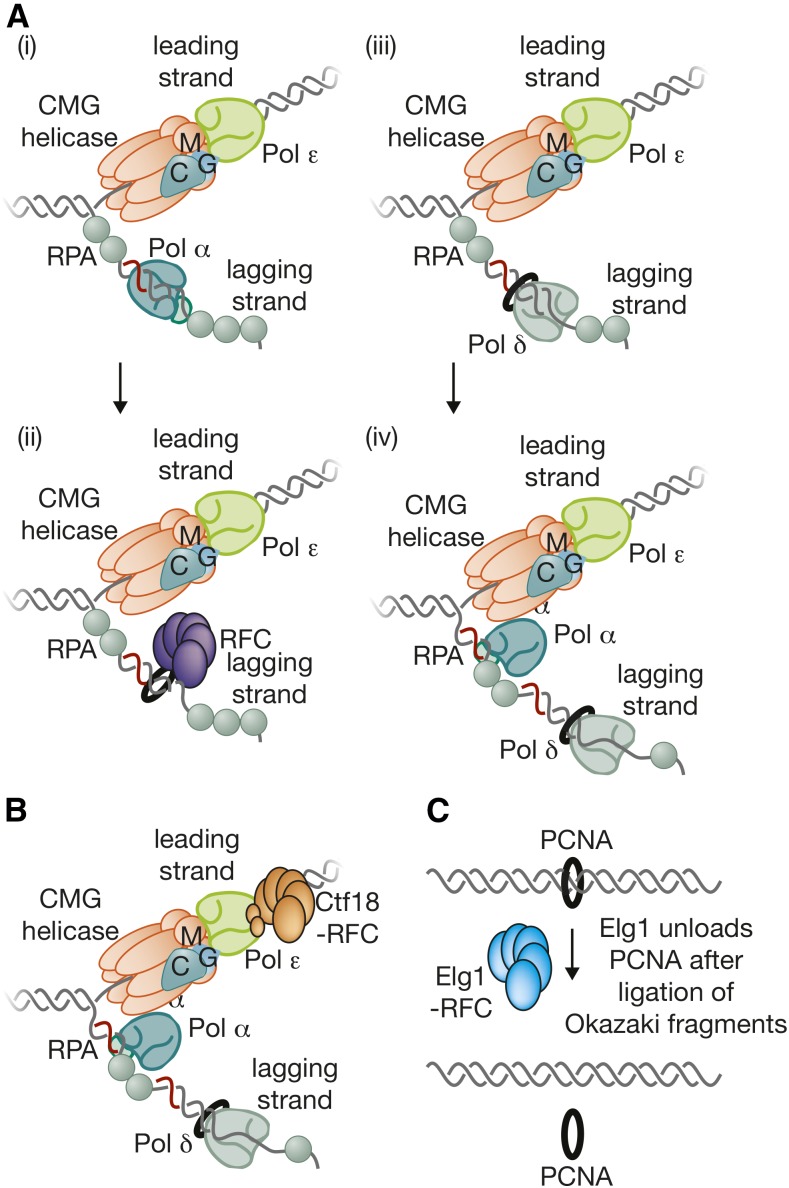
Multiple clamp loaders at the yeast replication fork. (A) Pol α detaches from the template after synthesizing an RNA-DNA primer (i), and Rfc1-RFC is then very effective at competing for access to the 3′ end of the primer bound to template, leading to loading of PCNA around dsDNA (ii). This in turn leads to recruitment of Pol δ (iii), which then extends the new Okazaki fragment (iv). (B) Ctf18-RFC associates with Pol ε and might contribute to loading of PCNA onto the leading-strand side of the fork. (C) Elg1-RFC is recruited to PCNA (aided by sumoylation) after ligation of Okazaki fragments, leading to removal of PCNA from the replicated DNA.

Replication Factor C (RFC; originally identified in SV40 replication studies) also competes for association with the 3′ end of RNA-DNA primers (Figure 8A). RFC is a pentameric complex of paralogous AAA+ proteins that is part of the family of clamp loaders (Yao and O’Donnell 2012). RFC breaks open the homotrimeric ring known generically as PCNA (proliferating cell nuclear antigen; the name comes from the human ortholog) and in budding yeast as Pol30. PCNA/Pol30 serves as a processivity factor for Pol δ and is loaded by RFC around dsDNA at primer-template junctions. PCNA then recruits Pol δ and clamps it tightly to its template, thus facilitating processive synthesis of Okazaki fragment DNA. The *in vitro* activity of yeast Pol δ is stimulated at least 100-fold by PCNA (Chilkova *et al.* 2007). Supporting Pol δ synthesis of the lagging strand; Pol δ outcompetes Pol ε *in vitro* for extension of a lagging-strand template that has been loaded with PCNA (Georgescu *et al.* 2014).

The role of PCNA in leading-strand synthesis remains unclear. In contrast to Pol δ, Pol ε is an inherently processive enzyme that tethers itself to the template by wrapping around dsDNA (Hogg *et al.* 2014). The *in vitro* activity of Pol ε in association with the CMG helicase is only stimulated twofold by PCNA (Georgescu *et al.* 2014). This result suggests that the processive action of Pol ε relies on its stable association with the CMG helicase and the DNA template.

## The CMG DNA Helicase Associates with Other Factors to Form the Replisome Progression Complex

When the CMG helicase (Moyer *et al.* 2006) was isolated from extracts of S-phase yeast cells (Gambus *et al.* 2006), mass spectrometry analysis indicated the presence of a specific set of associated factors (Figure 7B) forming a larger assembly known as the replisome progression complex (RPC) (Gambus *et al.* 2006). In addition to CMG, the RPC contains the Ctf4 adaptor protein; the type 1 topoisomerase Top1; the histone chaperone FACT; and the trimeric complex of regulatory factors comprising Tof1, Csm3, and the checkpoint mediator Mrc1. The various RPC components were shown by ChIP to migrate with replication forks (Aparicio *et al.* 1997; Kanemaki *et al.* 2003; Katou *et al.* 2003; Osborn and Elledge 2003; Takayama *et al.* 2003; Calzada *et al.* 2005; Gambus *et al.* 2006; Foltman *et al.* 2013) where they play diverse roles that are discussed in more detail in the following sections. Subsequently the RPC was also found to associate with additional factors (Figure 7C) including DNA polymerase α (Gambus *et al.* 2009), DNA polymerase ε (De Piccoli *et al.* 2012; Sengupta *et al.* 2013), and the E3 ubiquitin ligase known as SCF^Dia2^ (Morohashi *et al.* 2009) as discussed below.

## Tidying Up the Ends: Completing the Synthesis of Okazaki Fragments

**Figure 9:**
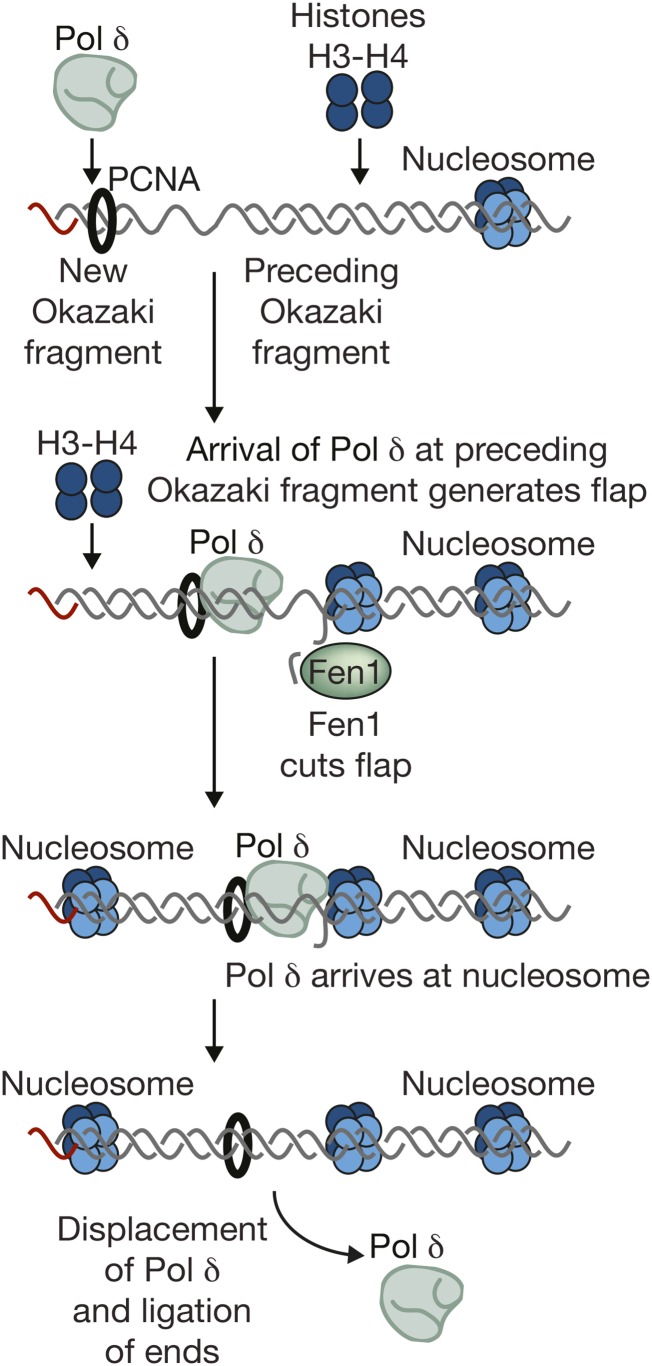
Processing of Okazaki fragments. The figure illustrates the model whereby nucleosome deposition plays a key role in completing the synthesis of Okazaki fragments. When Pol δ meets the 5′ end of the preceding Okazaki fragment, it displaces a short flap that is cut by Fen1 (or a longer flap that can be cut by Dna2). Strand displacement continues until Pol δ reaches the midpoint of the nucleosome deposited on the preceding fragment, at which point Pol δ detaches from the template, allowing ligation and thus completion of DNA synthesis.

When Pol δ extends a particular Okazaki fragment, it soon reaches the 5′ end of the preceding fragment, marked with an RNA primer (Figure 9). Instead of terminating at this point, Pol δ can continue DNA synthesis and displace part of the preceding Okazaki fragment in the form of a 5′ flap (Garg *et al.* 2004). This flap can be processed in a variety of ways before ligation of the remaining DNA ends by DNA ligase I. Short flaps are cleaved by an endonuclease known as Rad27/Fen1. Like Pol δ, Fen1 is recruited to replication forks by its interaction with PCNA (Li *et al.* 1995), in this case via a **P**CNA **i**nteracting **p**eptide (PIP box) in Fen1 (Gary *et al.* 1999). In contrast, longer flaps are cleaved preferentially by the nuclease activity of Dna2 (Bae *et al.* 2001; Ayyagari *et al.* 2003). Dna2 is normally essential *in vivo*, but becomes dispensable in cells lacking the Pif1 DNA helicase, probably reflecting the ability of Pif1 to load onto the 5′ end of Okazaki fragments and thus produce long flaps (Budd *et al.* 2006).

Genome-wide mapping of Okazaki fragments indicates that Pol δ often advances until it reaches the midpoint of a newly-formed nucleosome on the preceding Okazaki fragment (Smith and Whitehouse 2012). This finding led to a model in which nucleosomes trigger Pol δ release. In this model, the size of Okazaki fragments is not so much determined by the frequency of initiation events by Pol α, but instead by the spacing of nucleosomes; producing an average Okazaki fragment size that is close to the nucleosome repeat length of 165 bp (Smith and Whitehouse 2012). This mechanism for the processing of Okazaki fragments, based on the generation and subsequent cleavage of flaps, helps to preserve genome integrity, since the DNA synthesized by the error-prone Pol α is subsequently removed and then resynthesized by the much more reliable Pol δ. Nevertheless, it has been estimated that Pol α contributes up to 1.5% of the mature form of the replicated genome, perhaps representing those events where Pol δ meets the preceding Okazaki fragment and is released without generating a flap (Clausen *et al.* 2015; Reijns *et al.* 2015). This might occur when DNA binding proteins associate rapidly with a newly-synthesized Okazaki fragment, providing a barrier to the advancing Pol δ that would then be analogous to the nucleosome barrier described above. In addition, *in vitro* experiments indicate that replication in the absence of nucleosome assembly can still produce Okazaki fragments of approximately wild-type size (Georgescu *et al.* 2015).

## Breaking and Remaking Chromatin

**Figure 10:**
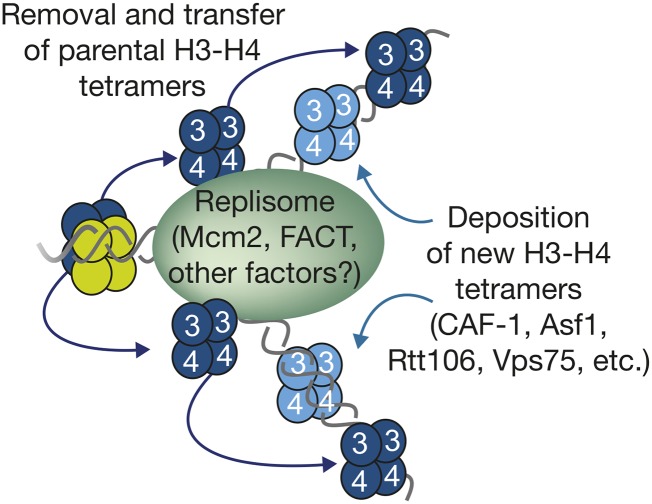
Regeneration of chromatin during DNA replication. DNA unwinding by the CMG helicase displaces parental histones, but it is thought that a tetramer of H3-H4 is retained locally, probably by the histone-binding activity of replisome components including Mcm2 and FACT. This allows for the local redeposition of parental H3-H4 tetramers onto the nascent DNA, in parallel with the deposition of newly-synthesized histones H3-H4 by chaperones such as CAF1. Following addition of H2A-H2B, nucleosomes are regenerated, and in practice this whole process is extremely rapid. It is assumed that epigenetic modifications on parental histones are then copied to the neighboring newly-synthesized nucleosomes, thus restoring parental chromatin.

Chromatin is both the substrate and the product of chromosome replication in eukaryotes (Figure 10). The phenomenal compaction of DNA into chromatin poses a significant challenge to the chromosome-replication machinery, which must “unpack” and disrupt chromatin at the replication fork to access the DNA template. At the same time, chromatin is reconstituted immediately behind the replication fork (Lucchini and Sogo 1995; Sogo *et al.* 2002; Whitehouse and Smith 2013) in such a way as to preserve epigenetic information and avoid disruption to the cellular program of gene expression.

### Disrupting parental chromatin at replication forks

At present it is unclear whether the replisome progresses through chromatin under its own steam, or whether additional factors are needed to disrupt chromatin immediately in front of DNA replication forks. Unwinding of the DNA template will displace histones and, thus, disrupt nucleosomes (Shundrovsky *et al.* 2006); but it is unknown whether the CMG helicase performs this task unassisted. Several chromatin remodeling enzymes such as Ino80 and Isw2 have been reported to play important roles during chromosome replication (Papamichos-Chronakis and Peterson 2008; Shimada *et al.* 2008; Vincent *et al.* 2008). Moreover, the histone chaperone FACT migrates with replication forks *in vivo* (Foltman *et al.* 2013) and is physically associated with the CMG helicase as part of the RPC (Gambus *et al.* 2006). However, the action of these factors at replication forks remains unclear.

### Preserving the status quo

The nascent DNA at replication forks must be repackaged very quickly into chromatin, not only to restore the normal density of nucleosomes, but also to preserve the parental pattern of epigenetic histone modifications. To achieve the latter, it is thought that parental histones from nucleosomes immediately in front of the replisome are distributed locally to both of the nascent DNA duplexes formed immediately behind the same replication fork (Radman-Livaja *et al.* 2011). The unit of transfer is likely to be a tetramer of histones H3 and H4, which carry the majority of epigenetic information and which do not appear to be disrupted by the DNA replication (Prior *et al.* 1980; Yamasu and Senshu 1990; Vestner *et al.* 2000; Katan-Khaykovich and Struhl 2011). The subsequent reassociation of H3-H4 tetramers with dimers of H2A-H2B would regenerate nucleosomes with similar properties to the parental chromatin before passage of the replication fork (Alabert and Groth 2012; Whitehouse and Smith 2013).

The mechanism of transfer of H3-H4 tetramers remains unclear. Passive transfer by diffusion cannot be ruled out, but seems a precarious way of preserving local patterns of epigenetic information. Alternatively, H3-H4 tetramers might be transferred actively by histone chaperones that are tethered to the chromosome-replication machinery. As discussed below, histone chaperones that build new chromatin during DNA replication bind to dimers of H3-H4, and structural information indicates that the interactions involve interfaces of H3 and H4 that are hidden within the H3-H4 tetramer (Antczak *et al.* 2006; English *et al.* 2006; Natsume *et al.* 2007). Thus, it is unclear how these chaperones could transfer intact tetramers of parental H3-H4 histones onto nascent DNA at replication forks. However, the replisome itself has histone-binding activity and thus could play a direct role in the transfer of parental H3-H4 tetramers. The Mcm2 subunit of the CMG DNA helicase has a conserved motif in its extended N-terminal tail (Foltman *et al.* 2013) that binds to parental histone complexes released from DNA (Foltman *et al.* 2013). Mutations of two conserved tyrosines in the Mcm2 tail abolish histone-binding activity (Foltman *et al.* 2013). Crystal structures of human Mcm2 tail bound to histones showed that these two conserved residues are key contact points with H3 and H4 (Huang *et al.* 2015; Richet *et al.* 2015). Moreover, the Mcm2 tail binds to the outside of the H3-H4 tetramer (Huang *et al.* 2015; Richet *et al.* 2015), analogous to the binding of DNA. Mutation of the histone-binding motif of Mcm2 in yeast cells does not affect DNA synthesis *per se*, but instead leads to a loss of subtelomeric silencing (Foltman *et al.* 2013); indicating a disruption of repressive chromatin at particular loci.

It is likely that the replisome will also contain other histone-binding activities that contribute to the transfer of parental histones during chromosome replication. The strongest candidate is FACT, which was first isolated as a partner of Pol α in budding yeast (Wittmeyer and Formosa 1997; Zhou and Wang 2004), and then found in human cells to be important for transcription through chromatin (Orphanides *et al.* 1998). FACT also associates with CMG as part of the RPC (Gambus *et al.* 2006; Foltman *et al.* 2013) and is able to cochaperone histone complexes with Mcm2 (Foltman *et al.* 2013). Mutations in FACT affect chromosome replication (Schlesinger and Formosa 2000), but this genetic analysis is complicated by FACT’s role in transcription, since defects in transcription can be an indirect source of replication defects.

It is also possible that Pol ε contributes to the regeneration of parental chromatin during the process of chromosome replication. Mutations in Pol2 or in the two histone-fold subunits of Pol ε, Dpb3, and Dpb4, cause defects in subtelomeric silencing (Iida and Araki 2004). The underlying mechanism remains unclear, but it is interesting that Dpb4 is also part of the chromatin remodeling complex known as the yeast chromatin **ac**cessibility complex (yCHRAC), in which it forms part of an analogous pair of histone-fold subunits with the **D**pb3**-l**ike **s**ubunit 1 (Dls1) protein. The histone-fold pair of such chromatin remodelers or transcription factors is thought to contribute to chromatin binding by association both with DNA and histones. The Dpb3-Dpb4 complex contributes to the ability of Pol ε to bind to dsDNA (Tsubota *et al.* 2006), together with Pol2 (Hogg *et al.* 2014), but a putative histone-binding activity for Pol ε remains to be explored.

### Building new nucleosomes

Although transfer of parental H3-H4 tetramers to nascent DNA at replication forks would help to preserve epigenetic information during chromosome replication, this process would halve the density of nucleosomes. Thus, there is also a requirement for the assembly of new nucleosomes during chromosome replication. This is an extremely-rapid process, since EM analysis of nucleosome density at replication forks indicates that nascent DNA at replication forks is already chromatinized to the same degree as the parental DNA (Lucchini and Sogo 1995; Sogo *et al.* 2002).

There is a large burst of histone synthesis during S phase, and the newly-synthesized histones are bound by a range of chaperones that contribute to the deposition of nascent histones onto DNA at replication forks. Others have reviewed this area extensively in the past (De Koning *et al.* 2007; Ransom *et al.* 2010; Alabert and Groth 2012; Amin *et al.* 2013) and here we will simply provide a summary in outline of the best-characterized pathway. The chaperone anti-silencing factor 1 (Asf1) binds to a dimer of newly-synthesized H3 and H4 (Antczak *et al.* 2006; English *et al.* 2006; Natsume *et al.* 2007), leading to acetylation of lysine 56 of H3 by Rtt109 (Masumoto *et al.* 2005; Schneider *et al.* 2006; Driscoll *et al.* 2007; Tsubota *et al.* 2007). Asf1 then passes the modified H3-H4 dimer (Li *et al.* 2008; Rolef Ben-Shahar *et al.* 2009) to another chaperone called chromatin assembly factor 1 (CAF1), which is recruited to nascent DNA at replication forks by its interaction with PCNA (Zhang *et al.* 2000; Li *et al.* 2008; Rolef Ben-Shahar *et al.* 2009). CAF1 is able to receive two H3-H4 dimers from Asf1 (Winkler *et al.* 2012), and is thus likely to plays a direct role in depositing a tetramer of H3-H4 on the nascent DNA, before other chaperones recruit dimers of H2A and H2B; leading to the formation of a new nucleosome. Note that neither Asf1 nor the components of CAF1 are essential for cell viability (Kaufman *et al.* 1997; Le *et al.* 1997) due to redundancy with other chaperones, such as Vps75 (Selth and Svejstrup 2007; Tsubota *et al.* 2007; Berndsen *et al.* 2008; Park *et al.* 2008) and Rtt106 (Huang *et al.* 2005; Li *et al.* 2008).

As described above, the nascent chromatin immediately behind replication forks will contain a mixture of nucleosomes with parental histones and their associated epigenetic marks, plus nucleosomes that are built entirely from newly-synthesized histones. It is thought that the epigenetic marks on parental histones recruit enzymes that add the same modifications to adjacent histones, propagating the modification to adjacent “virgin” nucleosomes. In this way, the epigenetic landscape of the newly-replicated chromatin can be restored to that of the parental template. This model remains speculative, and it is possible that many types of epigenetic information are reestablished *de novo* after replication.

### How is chromatin assembled on the leading-strand side of the fork?

A PCNA-dependent chromatin-assembly mechanism makes it easy to understand how chaperones such as CAF1 assemble new nucleosomes onto nascent lagging-strand DNA. This DNA is coated with multiple PCNA rings (recruiting CAF1 and other chaperones) due to the repeated cycles of RNA-DNA priming by Pol α, PCNA loading by RFC, and extension by Pol δ. This mechanism does not apply to the leading-strand DNA, however, where a single primer is extended from the origin. In principle, therefore, the newly-synthesized leading-strand DNA behind replication forks might be expected to lack PCNA compared to the nascent lagging strand. It is not yet clear how cells solve this conundrum, but one suggestion is that Pol ε promotes PCNA loading by RFC onto newly-synthesized leading-strand DNA, despite the apparent lack of new priming events (Chilkova *et al.* 2007; Georgescu *et al.* 2014; Kunkel and Burgers 2014). In favor of this idea, more PCNA accumulates on replicated DNA *in vitro* when a primed template is extended by Pol ε in the presence of RFC, compared to the equivalent reaction with Pol δ (Chilkova *et al.* 2007). Potentially, Pol ε detaches transiently from the end of the primer more frequently than what occurs during synthesis by Pol δ, providing transient access for RFC to load additional PCNA clamps (Georgescu *et al.* 2014; Kunkel and Burgers 2014). However, other repair mechanisms are also likely to contribute to PCNA loading onto leading-strand DNA (Lujan *et al.* 2013) and Pol ε associates with a specialized clamp loader known as Ctf18-RFC (Garcia-Rodriguez *et al.* 2015), which contributes to PCNA loading *in vivo* (Lengronne *et al.* 2006; Kubota *et al.* 2011) though *in vitro* studies have also highlighted the ability of Ctf18-RFC to unload PCNA from DNA (Bylund and Burgers 2005).

### Removing PCNA from nascent DNA behind replication forks

Despite the important role of PCNA on nascent DNA behind replication forks, it is also important that these clamps be removed from nascent chromatin to restore the pool of free PCNA for new replication forks or for DNA repair reactions. The removal of PCNA behind DNA replication forks is not well understood, but seems to involve the same family of RFC clamp loaders that are responsible for loading of PCNA at forks.

The RFC family all share the same core, comprising the Rfc2-5 subunits, but each complex uses a different paralog of Rfc1 as the largest subunit, which then confers specificity of action (Ulrich 2013). Rfc1-RFC and Ctf18-RFC are thought to act predominantly as loaders of PCNA *in vivo*, but a third member of the RFC family promotes the unloading of PCNA from replicated DNA *in vivo* (Figure 8C). The **e**nhanced **l**evels of **g**enome instability 1 protein (Elg1) is important for genome integrity, though it is not essential for cell viability in the laboratory (Kanellis *et al.* 2003). A proteomic study indicated that PCNA accumulates on chromatin in the absence of Elg1 (Kubota *et al.* 2011), and Elg1-RFC stimulates the release of PCNA from yeast chromatin *in vitro* (Kubota *et al.* 2013), analogous to the action of the human Elg1 ortholog known as ATAD5 (Lee *et al.* 2013). Moreover, PCNA unloading by Elg1 is linked to the ligation of Okazaki fragments (Kubota *et al.* 2015). It is also possible, however, that there is some degree of redundancy between RFC family members with regard to PCNA unloading.

## Controlling the Progression of Replication Forks

DNA replication forks must traverse the entirety of the genome during the process of chromosome replication. The task is aided by the activation of many origins on each chromosome, which reduces the distance that each individual fork needs to cover, and also provides important backup in case of problems at individual forks. Nevertheless, timely completion of replication requires that the rate of fork progression must be maintained at a high rate—about 1.5 kb per minute in yeast cells (Raghuraman *et al.* 2001; Yabuki *et al.* 2002; Sekedat *et al.* 2010)—and forks need to overcome a diverse range of obstacles as they travel from each origin to the point of termination. In addition to disrupting chromatin and displacing histones; forks must bypass many sites such as centromeres and transfer RNA (tRNA) promoters where nonnucleosomal proteins bind very tightly to DNA; cope with any DNA damage or unusual structures that might be generated in the unwound template; and deal with supercoils in the parental DNA ahead of the fork, which are generated by the action of the replicative helicase.

### Setting the rate of fork progression

Fork progression depends upon unwinding of the template DNA by the CMG helicase. However, the rate of progression of CMG is influenced by other replisome components, and in particular by the physical association of CMG with Pol ε (Georgescu *et al.* 2014). Pol ε but not Pol δ stimulates CMG activity *in vitro*, a feature that applies both to the yeast (Georgescu *et al.* 2014) and human (Kang *et al.* 2012) proteins. Stimulation of yeast CMG requires the Dpb2 subunit of Pol ε (Langston *et al.* 2014), which tethers Pol ε to CMG at forks (Sengupta *et al.* 2013). The mechanism is not known, but it is possible that Pol ε promotes a structural change in the CMG helicase that enhances activity of the latter. Alternatively, the polymerase activity of Pol ε might propel CMG forward or prevent the helicase from slipping backward on the unwound template DNA strand. This regulation would be analogous to the workings of the *E. coli* replisome, for which the rate of fork progression is set by the inherently fast rate of synthesis by the DNA polymerase, rather than by the inherently much-slower rate of unwinding by the DNA helicase (Kim *et al.* 1996).

Consistent with DNA polymerases setting the rate of progression of the DNA helicase at yeast replication forks, ChIP studies have shown that a reduction in the supply of dNTPs not only slows DNA synthesis, but also slows helicase progression to the same degree, indicating that the entire replisome moves slowly under such conditions (Aparicio *et al.* 1997; Kanemaki *et al.* 2003; Katou *et al.* 2003; Takayama *et al.* 2003). This regulation reduces the amount of ssDNA that would otherwise be exposed if the helicase were to continue at the same rate after the slowing of DNA synthesis.

Other replisome components can also influence the rate of fork progression, though the underlying mechanisms remain unclear. Forks move at about half their normal rate in the absence of Mrc1 (Szyjka *et al.* 2005; Tourriere *et al.* 2005; Hodgson *et al.* 2007). This effect is not seen in cells lacking the Rad53 checkpoint kinase (Versini *et al.* 2003), indicating that Mrc1 influences fork rate by a mechanism independent of its role in checkpoint signaling. A similar (Tourriere *et al.* 2005), though milder (Hodgson *et al.* 2007), reduction in fork rate is seen in the absence of Tof1 and Csm3, which tether Mrc1 to the replisome (Katou *et al.* 2003; Bando *et al.* 2009). Interestingly, Mrc1 associates both with Pol ε (Lou *et al.* 2008) and the Mcm6 subunit of the CMG helicase (Komata *et al.* 2009), but it is not yet known whether Mrc1 directly modulates the action of either component.

### Putting on the brakes

Two-dimensional DNA gels and ChIP have shown that replication forks pause at a variety of places around the genome. In particular, pausing occurs at sites where nonnucleosomal proteins are bound very tightly to DNA, including the ribosomal DNA (rDNA) (Brewer and Fangman 1988; Linskens and Huberman 1988; Calzada *et al.* 2005), centromeres (Greenfeder and Newlon 1992), tRNA promoters (Deshpande and Newlon 1996; Ivessa *et al.* 2003; De Piccoli *et al.* 2012), silent origins of replication (Wang *et al.* 2001), and telomeres (Makovets *et al.* 2004). Some barriers are unidirectional, such as tRNA promoters (Deshpande and Newlon, 1996; Ivessa *et al.* 2003) or the replication fork barrier that results from binding of the Fob1 protein to specific sequences in the rDNA repeats (Brewer and Fangman, 1988; Linskens and Huberman 1988). Others, such as centromeres, are able to pause forks that arrive from either direction (Greenfeder and Newlon 1992).

Interestingly, pausing at protein–DNA barriers is independent of Mrc1 (Calzada *et al.* 2005; Szyjka *et al.* 2005; Tourriere *et al.* 2005; Mohanty *et al.* 2006; Hodgson *et al.* 2007), but requires the Tof1-Csm3 complex (Calzada *et al.* 2005; Tourriere *et al.* 2005; Mohanty *et al.* 2006; Hodgson *et al.* 2007) that associates with the CMG helicase as part of the RPC (Gambus *et al.* 2006). These findings mirror earlier studies of fission yeast Swi1 and Swi3, orthologs of Tof1-Csm3; which are also required for forks to pause at protein–DNA barriers (Dalgaard and Klar 2000; Krings and Bastia 2004). Thus, pausing is not merely due to a replisome crashing into barriers that necessarily slow its progress, but instead represents an evolved feature of the replication machinery (Mayer *et al.* 2004; Warren *et al.* 2004). Pausing might allow other accessory factors to help remove the barriers (see below), before the brake is removed and the replisome resumes its progression. In other words, braking forks would be better than breaking forks, which might otherwise occur at a higher rate when forks pass through such barrier sites. However, the importance and molecular mechanism of pausing at protein–DNA barriers are not yet understood. Cells lacking Tof1 or Csm3 have enhanced rates of genome instability (Mayer *et al.* 2004; Warren *et al.* 2004), but it is unclear whether this is due to defective pausing of replication forks, or reflects other functions of Tof1-Csm3.

Other evidence suggests that the progression of the replisome can be slowed by an active signaling mechanism, in response to defects in replication that activate the protein kinases of the S-phase checkpoint pathway. In cells lacking protein phosphatases that dephosphorylate targets of the Rad53-checkpoint kinase, hyperactivity of Rad53 after DNA damage reduces fork speed (Szyjka *et al.* 2008). The underlying mechanism remains to be elucidated, but an interesting possibility would be that the CMG helicase, or key partners such as Pol ε, are regulated directly by phosphorylation under such conditions.

### Avoiding tangles

Unwinding of the parental DNA duplex at replication forks leads to the accumulation of positive supercoils in front of the fork, which would quickly inhibit helicase action and replisome movement if not removed. Topoisomerase I (Top1) and topoisomerase II (Top2) act redundantly to remove such supercoils (Bermejo *et al.* 2007). Top1 copurifies with the CMG helicase as part of the RPC (Gambus *et al.* 2006), suggesting that it is the primary topoisomerase acting at forks, reminiscent of the interaction of Top1 with T-antigen at the SV40 replication fork (Simmons *et al.* 1996). The link between Top1 and CMG is not understood, but an attractive possibility is that they are connected by the Tof1-Csm3 complex (Tof1 was identified as topoisomerase interacting factor 1). Interestingly, Tof1 is required to reduce fork rotation during elongation that would otherwise increase the number of precatenanes, which are double-stranded intertwines behind replication forks (Schalbetter *et al.* 2015). The mechanism is not known, but it is possible that Tof1 increases the local concentration of Top1 at forks. Alternatively, Tof1-Csm3 might be important for some aspect of replisome structure that reduces fork rotation. In contrast to Top1, Top2 is not part of the replisome but is a major chromatin-associated protein (Bermejo *et al.* 2009).

### CMG is not the only helicase

Budding yeast cells contain two members of the Pif1 family of DNA helicases that play important roles at DNA replication forks, without being essential for fork progression *per se*. These helicases, called Pif1 and Rrm3, have the opposite polarity to the CMG helicase (Lahaye *et al.* 1991; Ivessa *et al.* 2002) and help to unwind sites that might otherwise represent barriers for replisome progression. Two-dimensional DNA gel analysis has shown that Rrm3 is important to help forks pass through all classes of site where tight binding of nonnucleosomal proteins to DNA produces a “barrier” (Ivessa *et al.* 2003). In contrast, Pif1 plays a crucial role in disrupting DNA motifs in the genome that are prone to form G-quadruplex structures (Paeschke *et al.* 2011, 2013). These structures can impede the progression of the replisome and promote genome instability. Despite the apparent differences in the action of Pif1 and Rrm3, there is some functional redundancy (Paeschke *et al.* 2013). If the action of the CMG helicase in the replisome is akin to a high-speed train, it appears that Pif1 and Rrm3 are able to act like snow plows that clear away more troublesome barriers, thus enabling the CMG helicase to resume normal service.

Combined inactivation of the Sgs1 DNA helicase (ortholog of the human helicase mutated in Bloom’s syndrome) and the Srs2 helicase/translocase is lethal in yeast cells (Lee *et al.* 1999). This lethality was originally thought to reflect an essential role for Sgs1 and Srs2 in fork progression (Lee *et al.* 1999), but was subsequently shown to be due to excessive DNA recombination (Gangloff *et al.* 2000). It is now clear that Srs2 restrains recombination at replication forks (see below), whereas Sgs1 processes intermediates of DNA recombination reactions (Hickson and Mankouri 2011).

## Keeping Sisters Together

Following chromosome replication, each new pair of sister chromatids remains closely aligned with each other along their length. This process of cohesion is mediated at many points along the sister chromatids by a very large (100-nm diameter) proteinaceous ring called cohesin, within which the pair of chromatids are embraced (Uhlmann 2004; Nasmyth and Haering 2009; Oliveira and Nasmyth 2010; Marston 2014). By keeping the identical DNA sequences of the two sisters very close to each other, cohesion is critically important for pairs of sister chromatids to align properly on the metaphase spindle during mitosis and meiosis, and then segregate equally to different poles of the cell. In addition, cohesion facilitates DNA repair by homologous recombination (Klein *et al.* 1999; Sjogren and Nasmyth 2001).

Cohesin rings are loaded along the length of each chromosome before DNA replication and this sequence of events is very important for the subsequent establishment of cohesion, which is normally coupled to the passage of replication forks (Uhlmann and Nasmyth 1998). The molecular details of cohesion establishment during S phase are still poorly understood, and it is not clear what happens next when a replication fork meets a cohesin ring that has already been loaded around dsDNA. The simplest possibility would be that the replisome passes through the center of the cohesin ring without the latter needing to open, since this would ensure that the two sister chromatids are always trapped within the same set of cohesin rings along their length (Haering *et al.* 2002). Alternatively, the cohesin ring might open transiently to allow the replisome to pass by, before closing specifically around the two sister chromatids behind the fork.

### PCNA recruits the Eco1 enzyme that acetylates cohesin

The Eco1/Ctf7 protein is the only protein that is known to be essential for the establishment of cohesion during S phase, without also being required subsequently to maintain cohesion before mitosis (Skibbens *et al.* 1999; Toth *et al.* 1999). Growth defects produced by mutations in the *ECO1* gene can be suppressed by overexpression of the *POL30* gene that encodes PCNA, suggesting a link between Eco1 function and replication forks (Skibbens *et al.* 1999). Moreover, Eco1 has a PIP box that mediates its interaction with Pol30, and mutation of *POL30* also produces defects in cohesion establishment (Moldovan *et al.* 2006). Similar defects are observed in the absence of the Ctf18-RFC clamp-loader complex that is thought to contribute to loading of PCNA at replication forks (Hanna *et al.* 2001; Mayer *et al.* 2001; Lengronne *et al.* 2006), and *ctf18* mutations cause synthetic lethality in combination with mutations in *POL30* or *ECO1* (Skibbens *et al.* 1999; Collins *et al.* 2007). Overall, these data suggest that Eco1 is recruited by PCNA to replication forks, where it plays an essential role in establishing cohesion during chromosome replication.

Eco1 is an acetyltransferase that modifies specific sites in the Smc3 subunit of cohesin during S phase (Ben-Shahar *et al.* 2008; Unal *et al.* 2008; Zhang *et al.* 2008; Rowland *et al.* 2009). Acetylation counteracts the destabilizing effect of the Rad61/Wpl1 (the yeast ortholog of the mammalian destabilizer of cohesin called Wapl) protein upon the cohesin ring, and deletion of Rad61/Wpl1 suppresses the lethality of *eco1∆* (Ben-Shahar *et al.* 2008; Unal *et al.* 2008; Rowland *et al.* 2009). The same is true of mutations Pds5 and Scc3, which form a complex together with Rad61/Wpl1 that serves to destabilize cohesin *in vivo* (Rowland *et al.* 2009). Thus, stabilization of the cohesin ring during S phase is critical for cohesion to be established.

Surprisingly, it is possible to establish cohesion even after the end of chromosome replication by overexpression of Eco1 (Strom *et al.* 2007; Unal *et al.* 2007), the level of which normally drops after S phase (Lyons and Morgan 2011). So although the recruitment of Eco1 to replication forks by PCNA is normally a crucial aspect of cohesion establishment during S phase, the replication machinery is not otherwise required for Eco1 function.

### A second pathway for cohesion establishment at DNA replication forks?

Deletion of *CTF18*, *MRC1*, *TOF1*, or *CSM3* produces a cohesion defect that is reduced by additional removal of *WPL1* (Borges *et al.* 2013), indicating that these factors are important for the Eco1 pathway of cohesin acetylation. In contrast, deletion of *WPL1* does not suppress the cohesion defects of cells lacking either Ctf4 or the DNA helicase Chl1 (Skibbens 2004; Hanna *et al.* 2001; Mayer *et al.* 2004; Lengronne *et al.* 2006; Borges *et al.* 2013), suggesting that these factors contribute in a different way to cohesion establishment. The cohesion defect of *ctf4∆* is epistatic with that of *chl1∆* (Borges *et al.* 2013), and recent work has shown that Ctf4 recruits the Chl1 helicase to replication forks (Samora *et al*. 2016); although the mechanism by which Chl1 contributes to cohesion establishment remains unclear.

## Surviving DNA Replication

The need to unwind and duplicate every single base pair in the genome during chromosome replication provides a huge potential for mutations or the generation of chromosomal breaks and rearrangements. Cells are only able to survive chromosome replication due to the evolution of a complex array of pathways that monitor and then respond to defects in DNA synthesis. These systems allow cells to detect errors that are made during replication, or abnormalities in the progression of DNA replication forks, and then correct the mistakes and repair any DNA damage, as well as preserving the functional integrity of the replisome at replication forks. The various pathways have been summarized in many other reviews (Friedel *et al.* 2009; Segurado and Tercero 2009; Zegerman and Diffley 2009; Branzei and Foiani 2010; Ulrich and Walden 2010; Labib and De Piccoli 2011; Finley *et al.* 2012; Boiteux and Jinks-Robertson 2013; Zou 2013; Hills and Diffley 2014) and the following section simply provides an overview, highlighting areas that are still understood poorly.

### Avoiding errors during DNA synthesis

If either Pol ε or Pol δ incorporate the wrong nucleotide at replication forks, the polymerases themselves are often able to repair the resulting mismatch, using their proofreading exonuclease activity to cleave the phosphodiester bond that links the last nucleotide to the growing chain (Kunkel 2011). Cleavage produces a 3′ OH at the end of the nascent DNA molecule, and so is compatible with the continuation of DNA synthesis. Similarly, the importance of proofreading helps explain why Pol α/δ/ε are only able to extend preexisting chains (RNA in the case of Pol α and DNA in the case of Pol δ/ε), since the first nucleotide to be added cannot be proofread and thus would be a source of errors. Cells avoid this issue by using RNA primers that can subsequently be removed.

Any mistakes that escape the proofreading machinery are then corrected behind replication forks by the mismatch-repair system, which has been reviewed extensively in another chapter in this series (Boiteux and Jinks-Robertson 2013). The mismatched base pair produces a distortion in the double helix, which is recognized by heterodimeric complexes of Msh2 with Msh3 or Msh6. The newly-synthesized strand containing the mismatched base must become the substrate for repair. The mechanism of strand discrimination is best understood in some bacteria where DNA methylation plays an important role (Kunkel and Erie 2005; Putnam 2016), whereas in eukaryotes the presence of nicks is likely to be a key requirement. During synthesis of the lagging strand, such nicks are present at the termini of Okazaki fragments before processing is completed. A different mechanism applies to the leading strand, where ribonucleotides are incorporated with a low frequency by Pol ε, leading to excision by RNase H and the transient generation of nicks (Lujan *et al.* 2013). RNase H is important for mismatch repair, particularly on the leading-strand side of the fork (Ghodgaonkar *et al.* 2013; Lujan *et al.* 2013). Interestingly, Pol2 has retained a key residue in its active site, Met644, that predisposes Pol ε to incorporate ribonucleotides at a higher rate than is the case for Pol δ (Nick McElhinny *et al.* 2010a). Mutation of Met644 to Leu644 (the equivalent residue found in Pol3) reduces ribonucleotide incorporation (Nick McElhinny *et al.* 2010a), indicating that increased ribonucleotide incorporation by Pol ε has been selected for during evolution, likely due to its role in mismatch repair. Incidentally, this notion further supports the idea that Pol ε is indeed the leading-strand polymerase at replication forks.

After recognition of the mismatch, the complex of heterodimeric MutS is then joined by a second heterodimeric MutL complex comprising orthologs of bacterial MutL; with Mlh1 associated with Pms1, Mlh2, or Mlh3. It is thought that these MutL-related complexes introduce an additional nick in the newly-synthesized strand, on the opposite side of the mismatch to the original nick, thus creating a stretch of DNA that can be removed by helicase activity and then resynthesized by Pol δ before ligation.

### Surviving defects in DNA replication: the S-phase checkpoint pathway

**Figure 11:**
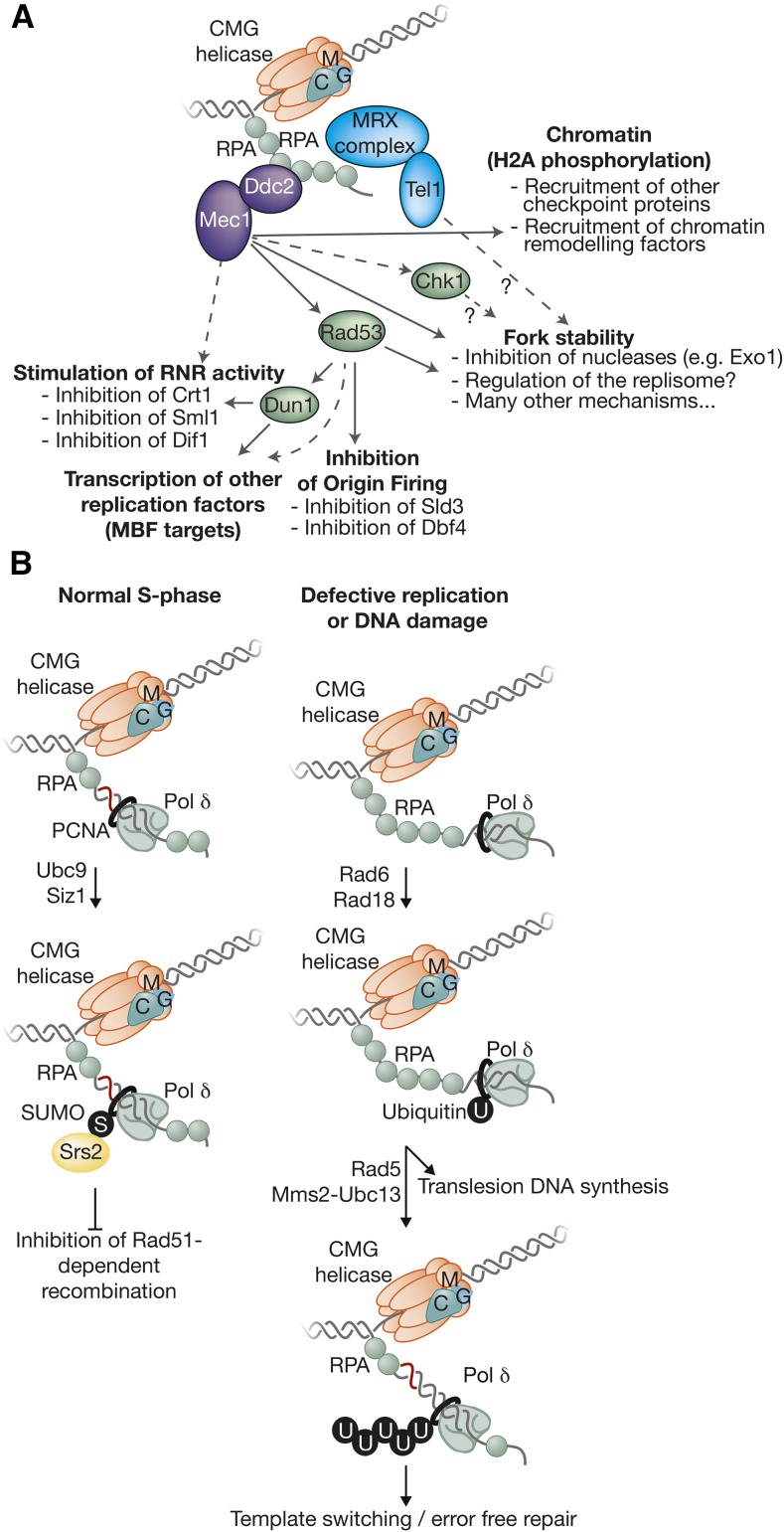
Surviving problems during chromosome replication. Replication defects expose more ssDNA at forks and thus lead to an accumulation of RPA. (A) This recruits Mec1-Ddc2 to initiate the S-phase checkpoint pathway and also (B) leads to ubiquitylation of PCNA, which activates translesion DNA synthesis and also an error-free repair pathway.

Defects in DNA synthesis at replication forks lead to a small accumulation of ssDNA (Sogo *et al.* 2002), which is rapidly coated by RPA (Figure 11A). This effect can be caused in the laboratory by a reduction in dNTP levels, for example by treating cells with hydroxyurea that inhibits ribonucleotide reductase. The accumulation of RPA-coated ssDNA provides the signal for the recruitment of the Mec1 checkpoint kinase to the defective replication fork, via its Ddc2/Lcd1 subunit that binds to RPA (Paciotti *et al.* 2000; Rouse and Jackson 2002; Zou and Elledge 2003). This interaction allows Mec1 to be activated by unstructured motifs in one of several replication factors (Zou 2013). Mec1 then phosphorylates a range of targets, including the replisome component Mrc1 that recruits the downstream checkpoint kinase Rad53. Recruitment of Rad53 promotes its autophosphorylation and activation (Alcasabas *et al.* 2001; Osborn and Elledge 2003). However, much remains to be learned about the mechanism of Rad53 activation, which also requires Ctf8-RFC and Pol ε (Crabbe *et al.* 2010; Kubota *et al.* 2011). It is possible that Pol ε forms a platform for checkpoint activation, since Rad53 activation is dependent upon the interaction of Ctf18-RFC with Pol ε (Garcia-Rodriguez *et al.* 2015), and Mrc1 also interacts with Pol ε (Lou *et al.* 2008).

Mec1 and Rad53 drive a number of responses that protect cells with stalled DNA replication forks. The first response to be characterized was that Mec1 and Rad53 block mitosis in response to replication defects (Weinert, 1992; Allen *et al.* 1994; Kato and Ogawa 1994; Weinert *et al.* 1994). But the checkpoint kinases also play many other important roles, including among others the stimulation of ribonucleotide reductase activity (Elledge *et al.* 1992; Zhou and Elledge 1993; Huang *et al.* 1998; Zhao *et al.* 1998; Lee *et al.* 2008; Bruin 2009), maintaining transcription of factors expressed during S phase (Bastos de Oliveira *et al.* 2012; Travesa *et al.* 2012), inhibition of the initiation factors Sld3 and Dbf4 at replication origins so that new forks are not generated until the source of the original defect has been removed (Santocanale and Diffley 1998; Shirahige *et al.* 1998; Lopez-Mosqueda *et al.* 2010; Zegerman and Diffley 2010), and the phosphorylation of histone H2A so as to recruit chromatin remodeling enzymes to the vicinity of replication forks (Downs *et al.* 2000; van Attikum and Gasser 2009). Not surprisingly, cells lacking Mec1 or Rad53 are exquisitely sensitive to DNA damage and other defects during chromosome replication (Weinert 1992; Allen *et al.* 1994; Kato and Ogawa 1994; Weinert *et al.* 1994).

In the absence of checkpoint kinases, DNA replication forks are unable to recover from “replication stress” and cannot resume DNA synthesis (Desany *et al.* 1998; Tercero and Diffley 2001). The reasons are likely to be many and varied, and the following discussion is certainly not exhaustive. There is evidence that the checkpoint helps to restrain the activity of certain nucleases at defective replication forks (though checkpoint kinases also activate other nucleases), which might otherwise induce further DNA damage (Segurado and Diffley 2008; Alabert *et al.* 2009). The replisome itself is also likely to be an important target for checkpoint kinases (Randell *et al.* 2010; De Piccoli *et al.* 2012). As discussed above, there is evidence suggesting that the checkpoint pathway restrains the progression of replication forks (Szyjka *et al.* 2008). Several subunits of the CMG helicase are targets of Mec1 (Randell *et al.* 2010; De Piccoli *et al.* 2012), though the functional significance of these modification remains to be explored. It seems very likely that our current understanding of checkpoint kinases at replication forks is only the tip of the iceberg.

Analyzing the many facets of the S-phase checkpoint is a considerable challenge. The multiple targets of Rad53 and Mec1 suggest that mapping and mutating phosphorylation sites in individual targets is unlikely to produce the dramatic phenotypes that are seen in cells that lack the checkpoint kinases. In theory, it might ultimately be possible to combine in a single cell a set of mutations in all the various target proteins, to recapitulate the phenotype of *mec1∆* or *rad53∆* cells, but the required number of mutations would probably be very high. For example, mutation of 19 serines or threonines in the Dbf4 protein was required to prevent inhibition by Rad53 (Zegerman and Diffley 2010).

### Ubiquitin and SUMO control important DNA damage responses during S phase

In addition to activating the S-phase checkpoint pathway, the accumulation of RPA-coated ssDNA at defective replication forks also recruits the Rad18 E3 ubiquitin ligase (Figure 11B), which activates another important branch of the DNA damage response (Ulrich and Walden 2010; Finley *et al.* 2012; Boiteux and Jinks-Robertson 2013). Rad18 promotes the monoubiquitylation of lysine 164 of PCNA by the Rad6 ubiquitin-conjugating enzyme (Hoege *et al.* 2002), which in turn leads to the recruitment of translesion DNA polymerases (Stelter and Ulrich 2003) such as Pol η (Rad30) and Pol ζ (Rev3-Rev7-Pol31-Pol32; Rev, **rev**ersionless). Unlike Pol ε or Pol δ, these polymerases are able to incorporate dNTPs opposite damaged bases. Although mutagenic, the translesion polymerases allow the replication machinery to bypass the damaged base, which can hopefully be repaired postreplicatively. Alternatively, monoubiquitylated PCNA can be modified further by the Rad5 ubiquitin ligase in association with the E2 complex called Mms2-Ubc13, producing a K63-linked ubiquitin chain at Lys164 of PCNA (Parker and Ulrich 2009). This modification activates a poorly understood error-free pathway of DNA repair. Interestingly, although the Rad6 pathway normally functions during S phase, it does not require ongoing DNA replication and can also act after chromosome replication has been completed (Daigaku *et al.* 2010).

PCNA is also sumoylated on Lys164 during an unperturbed round of DNA replication by the Siz1 E3 ligase in association with the Ubc9 SUMO-conjugating enzyme (Hoege *et al.* 2002). Sumoylated PCNA recruits the Srs2 translocase (Papouli *et al.* 2005; Pfander *et al.* 2005), which is thought to displace recombination factors and thus reduce illicit recombination events that might otherwise interfere with the action of DNA replication forks.

Finally, it seems clear that other E3 ubiquitin and SUMO ligases act to preserve genome integrity at DNA replication forks, though the relevant substrates remain to be identified. For example, the Rtt101 cullin (Scholes *et al.* 2001) forms an E3 ubiquitin ligase (Zaidi *et al.* 2008) analogous to the SCF, and cells lacking Rtt101 have an enhanced rate of genome instability and are sensitive to agents that perturb chromosome replication (Luke *et al.* 2006). In addition, Smc5 and Smc6 participate in a large complex analogous to cohesin and condensin, which is unique in having an associated SUMO ligase and ubiquitin ligase (Zhao and Blobel 2005). Smc5-Smc6 is clearly important for the preservation of genome integrity during chromosome replication (Branzei *et al.* 2006; Choi *et al.* 2010), particularly during the replication of large chromosomes where it might help to resolve topological problems at replication forks (Kegel *et al.* 2011), but much remains to be learned about its mechanism of action and its regulation.

## The End of the Road: Terminating DNA Replication

**Figure 12:**
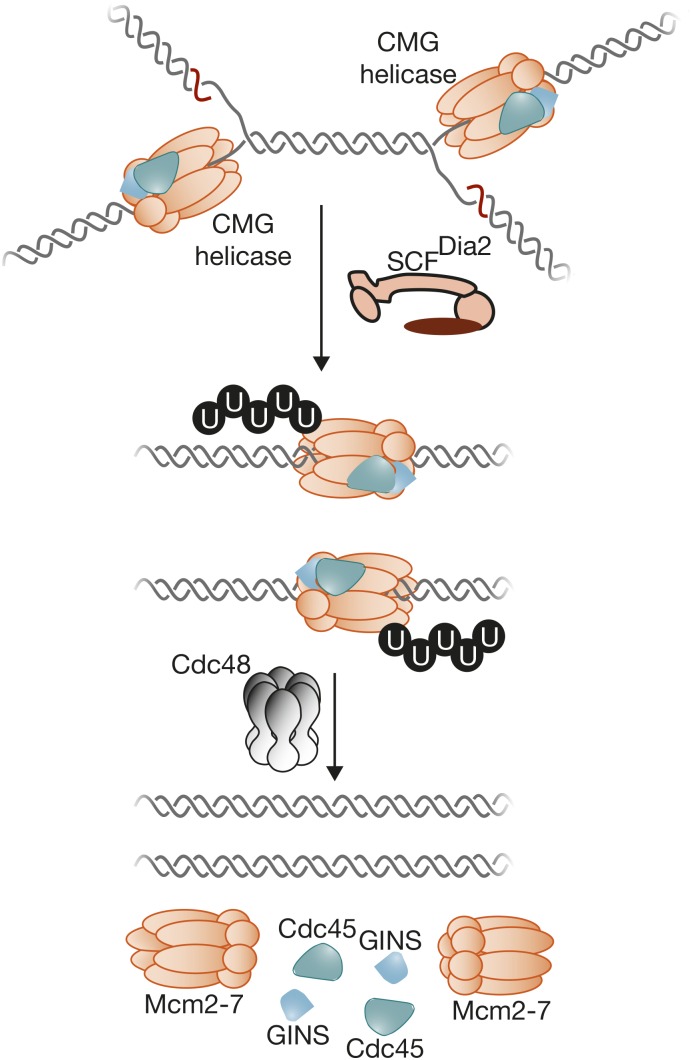
Disassembly of the CMG helicase is the final step in chromosome replication. When two forks converge, a poorly-characterized signal leads to ubiquitylation of the Mcm7 subunit of CMG, which is dependent upon the E3 ligase SCF^Dia2^. The Cdc48 segregase is then required for disassembly of ubiquitylated CMG, by a mechanism that is not yet understood.

Each DNA replication fork starts its journey at an origin and ends when it meets an opposing fork from a neighboring origin. Before the convergence of two replication forks, they must each continue progression past whatever obstacles are met along the way, to ensure that replication of the genome is completed. In particular, it is crucial that the CMG helicase is not lost from replication forks at any point during the elongation phase (Labib *et al.* 2000), as CMG represents the stable core around which the replisome is built, and the helicase cannot normally be reloaded during S phase (as discussed above). Nevertheless, the encounter of two replication forks always leads to the termination of DNA synthesis and to the rapid disassembly of the two replisomes at the converged forks. By analogy with the initiation reaction, disassembly of the CMG helicase should be the key regulated step in replisome dissolution, and helicase disassembly must be regulated to ensure that it never occurs prematurely. At present, the termination of DNA replication is understood much less well than the earlier stages of chromosome replication (Figure 12).

### Where to end?

Whereas origins of DNA replication occur at specific loci in the budding yeast genome; the sites where replication terminates are more stochastic, since termination will occur whenever and wherever two converging replication forks meet each other (or when a fork reaches the end of the chromosome). In principle, barriers to the progression of replication forks could also become site-specific termination sites, if pausing of one fork persists until arrival of the converging fork from a neighboring origin. This is an important mechanism in the rDNA repeats (Brewer and Fangman 1988; Linskens and Huberman 1988), where binding of Fob1 to the replication fork barrier provides a robust block to fork progression (Kobayashi and Horiuchi 1996). This barrier ensures that the ribosomal RNA (rRNA) genes are replicated by a fork that passes in the same direction as transcription, preventing repeated head-on collisions with the highly active rRNA transcription machinery. Elsewhere in the genome, termination zones also contain elements that pause replication forks, either in the form of centromeres or due to a clash with RNA polymerase II or III (Fachinetti *et al.* 2010). But genome-wide analysis of termination sites; by strand-specific sequencing of Okazaki fragments, or by deep sequencing to monitor DNA copy number change; indicates that the contribution of pausing elements to the determination of termination sites is relatively minor (Smith and Whitehouse 2012; Hawkins *et al.* 2013; McGuffee *et al.* 2013). Instead, the main factors that determine the sites of termination are the location and relative time of initiation of two adjacent initiation sites (Smith and Whitehouse 2012; Hawkins *et al.* 2013; McGuffee *et al.* 2013). If two neighboring origins fire at the same time as each other, termination will tend to occur at the midpoint between the origins, regardless of whatever pausing elements might be located between the origins.

### Removing tangles and other barriers to fork convergence

As discussed above, the action of topoisomerases I and II is required to remove positive supercoils from in front of replication forks and this is important to allow the continued unwinding of the parental DNA template by the CMG helicase (Bermejo *et al.* 2007). Topoisomerase activity is particularly important when two replication forks converge during the termination of DNA replication, as seen previously in studies of SV40 DNA replication (Sundin and Varshavsky 1980). Whereas it is clear that yeast Top1 and Top2 act in a redundant fashion during elongation, there is some evidence for a more-specific role for Top2 during termination (Fachinetti *et al.* 2010). The completion of DNA synthesis in termination zones is delayed in *top2-1* cells at the restrictive temperature of 37° (Fachinetti *et al.* 2010). Similarly, termination of plasmid DNA replication is delayed by a catalytically dead form of Top2, following depletion of degron-tagged wild-type Top2 that otherwise kept the cells alive (Baxter and Diffley 2008). Interestingly, however, depletion of Top2 did not by itself prevent the completion of DNA synthesis, though it did cause entanglement of the replicated sister chromatids, and thus led to DNA damage during chromosome segregation (Baxter and Diffley 2008). Thus, it appears that the presence of inactive Top2 protein can interfere with the convergence of two replication forks during termination; but Top2 is not necessarily essential for replication termination *per se*, presumably due to the compensating ability of Top1 to remove supercoils from between the two converging forks. Within the rDNA repeats, the convergence of DNA replication forks at the replication fork barrier is delayed in the absence of the Rrm3 DNA helicase (Ivessa *et al.* 2000), indicating that Rrm3 is also important for efficient termination, at least at certain protein–DNA barriers.

### The F-box protein Dia2 is essential for CMG disassembly at the end of chromosome replication

The F-box protein Dia2 is important to preserve genome integrity during chromosome replication (Pan *et al.* 2006) and forms the substrate-binding component of the E3 ubiquitin ligase known as SCF^Dia2^. Dia2 is essential for disassembly of the CMG helicase during completion of replication (Maric *et al.* 2014). As part of the disassembly process, CMG is ubiquitylated on its Mcm7 subunit in an SCF^Dia2^-dependent manner (Maric *et al.* 2014). SCF^Dia2^ is tethered to the RPC by a tetratricopeptide-repeat domain at the N-terminal of Dia2 which binds both Ctf4 and Mrc1 (Morohashi *et al.* 2009). This tethering mechanism increases the efficiency of CMG ubiquitylation and disassembly at the end of chromosome replication (Maculins *et al.* 2015).

### The Cdc48 ATPase is required to disassemble ubiquitylated CMG helicase

Inactivation of the Cdc48 segregase leads to accumulation of ubiquitylated CMG helicase on chromatin when cells progress through replication (Maric *et al.* 2014). The signal for ubiquitylation remains to be determined, but work with frog egg extracts indicates that CMG disassembly is likely to represent the final step in chromosome replication (Dewar *et al.* 2015). CMG disassembly would thus occur at different moments during S phase across the genome, once synthesis of each particular replicon has been completed.

Much remains to be learned about the mechanism of the reactions by which SCF^Dia2^ drives ubiquitylation of CMG helicase, before Cdc48-dependent disassembly. Ubiquitylation of Mcm7 correlates with recruitment of Cdc48 and rapid disassembly of the CMG helicase (Maric *et al.* 2014), but it still remains to be demonstrated that ubiquitylation of Mcm7 is essential for helicase disassembly. Another important issue is how helicase disassembly is restricted to terminating CMG complexes. One interesting possibility would be that the CMG helicase is altered when two forks converge; perhaps by a structural change in the Mcm2-7 ring, which then makes it accessible to SCF^Dia2^ and Cdc48. This begs the question of what happens at the telomeres (Wellinger and Zakian 2012), where termination only involves a single replication fork that reaches the end of the chromosome. Perhaps the helicase simply slides off the end of the template strand, inducing a similar structural change to that envisaged above.

## Perspectives

Budding yeast origins are unusual in requiring specific DNA sequence elements. Nevertheless, almost all the protein components of the yeast replication machinery have a single ortholog in humans and other species, and chromosome replication is one of the most highly-conserved areas of eukaryotic cell biology. Budding yeast still has much to offer for the future and should continue to drive our understanding of this fascinatingly-complex process in all eukaryotes.

The obsession of eukaryotic cells with replicating their chromosomes exactly once per cell cycle has put the loading of Mcm2-7 and formation of the CMG helicase into the spotlight over the last decade. This complex likely represents the most complicated helicase in biology, and important questions remain about how it is loaded and activated during the initiation of chromosome replication. Although we know more about helicase loading than any other step during replication initiation, fundamental questions still remain. How is the opening and closing of the Mcm2-7 ring controlled during helicase loading? Where does helicase loading occur relative to ORC and how is this process influenced by local chromatin structures? There are even more unknowns about the mechanisms of helicase activation. How do Cdc45 and GINS activate the Mcm2-7? How does the CMG transition from surrounding dsDNA during G_1_ to encircling ssDNA during elongation? What is the mechanism of initial DNA melting during this transition? What events drive the separation of the double hexamer formed during loading to the individual Mcm2-7 complexes involved in the elongating replisome/RPC? Finally, we know very little about the action and regulation of CMG during elongation. Do the DNA polymerases or other proteins modulate CMG function? Do checkpoint proteins modulate CMG activity?

Little is known about the architecture of the replisome that is built around the CMG helicase at replication forks, despite the fact that we probably know almost all the components of this complex machine. Many eukaryotic replisome subunits lack equivalents in bacteria, and remain of unknown function. Structural biology will have much to offer in future studies of the replisome, with improved resolution of replication complexes by EM complementing crystal structures of individual components. Single-molecule studies will also play an important role, allowing direct visualization of elongating replisomes and revealing the dynamic functions of the proteins involved.

Much remains to be learned about the biology of the events beyond DNA replication that are stimulated by the replisome. Duplicating a chromosome involves much more than just copying DNA, and the complexity of the eukaryotic replisome reflects the need to couple many other processes to DNA synthesis. We still know little about how the replication machinery traverses nucleosomes and reconstitutes chromatin during replication. How do replication forks transfer parental H3-H4 histone complexes onto nascent DNA? What is the importance of such mechanisms for the preservation of epigenetic histone marks during replication? The establishment of sister chromatid cohesion is also coupled to replication fork passage but the mechanisms that couple these two events are unknown. Finally, the many pathways that preserve genome integrity at DNA replication forks, both in the absence and presence of DNA damage, remain poorly understood.

The mechanism and regulation of replication termination remain unclear and represent another major area for future work. CMG disassembly is a key step and must not occur prematurely. At present it is not known whether termination involves other unique steps in addition to helicase disassembly; or if it involves processes that are important for fork progression throughout elongation, but that become more critical during the final stages of replication.

In all these important areas of study, a major challenge will be to develop *in vitro* systems with which to reconstitute each step, ultimately with purified components. Though a huge challenge, most of the events of initiation have already been reconstituted using budding yeast proteins, as have several key features of the elongation machinery. Development of assays that fully reconstitute a complete round of replication including full replisome assembly and termination remains elusive. In addition, most of the current *in vitro* assays for DNA replication events are performed in the absence of nucleosomes. It will be critical to extend these assays to nucleosomal templates to understand fully how replication initiation and elongation occur. Development of these and other assays will be critical to answer questions about the functions of specific proteins and the molecular events that they stimulate.

Dating back to the discovery of the double helix, chromosome replication is one of the oldest fields in molecular biology. Much has been learned over the last two decades, but the insights gained have highlighted how many fundamental questions remain unanswered. This is clearly just the beginning.
